# Regional disparities in blood pressure control after hypertension treatment initiation in Japan: a real-world data analysis

**DOI:** 10.1038/s41440-025-02454-y

**Published:** 2025-11-18

**Authors:** Yutaro Iwabe, Michihiro Satoh, Hiroki Nobayashi, Seiya Izumi, Takahisa Murakami, Maya Toyama, Takahito Yagihashi, Yuya Suzuki, Tomoko Muroya, Shingo Nakayama, Takayoshi Ohkubo, Hirohito Metoki

**Affiliations:** 1 Center for Clinical Research Promotion and Development, Tohoku Medical and Pharmaceutical University Hospital, Sendai, Japan; 2 Division of Public Health, Hygiene and Epidemiology, Faculty of Medicine, Tohoku Medical and Pharmaceutical University, Sendai, Japan; 3 Department of Pharmacy, Tohoku Medical and Pharmaceutical University Hospital, Sendai, Japan; 4 Department of Preventive Medicine and Epidemiology, Tohoku Medical Megabank Organization, Tohoku University, Sendai, Japan; 5 Division of Nephrology and Hypertension, Department of Internal Medicine, The Jikei University School of Medicine, Tokyo, Japan; 6 Department of Obstetrics and Gynecology, Tohoku University Graduate School of Medicine, Sendai, Japan; 7 Department of Nephrology, Self-Defense Forces Sendai Hospital, Sendai, Japan; 8 Department of Neurology, Faculty of Medicine, Tohoku Medical and Pharmaceutical University, Sendai, Japan; 9 Division of Nephrology and Hypertension, Tohoku Medical and Pharmaceutical University, Sendai, Japan; 10 Division of Internal Medicine, Izumi Hospital, Sendai, Japan; 11 Nanatsumori Family Clinic, Miyagi, Japan; 12 Department of Pathology and Laboratory Medicine, Cedars-Sinai Medical Center, Los Angeles, CA USA; 13 Department of Hygiene and Public Health, Teikyo University School of Medicine, Tokyo, Japan; 14 Tohoku Institute for Management of Blood Pressure, Sendai, Japan

**Keywords:** Blood pressure, Clinical inertia, Healthcare disparities, Digital hypertension, Implemental hypertension

## Abstract

This study assessed regional variations in blood pressure (BP) control after antihypertensive treatment and explored the associations with healthcare resource indicators across Japan. Using nationwide health check-up data from the Japan Health Insurance Association between 2015 and 2022, we analyzed 1,318,437 individuals aged 40–74 who initiated antihypertensive treatment based on consecutive health check-ups. We evaluated prefecture-level differences in post-treatment BP control rates (systolic BP [SBP]/diastolic BP [DBP] <130/<80 mmHg). Prefecture-level ecological analyses examined the associations between adjusted BP control rates and cerebrovascular disease mortality rates and six healthcare resource indicators, including the Physician Uneven Distribution Index (PUDI). Mean SBP/DBP decreased from 148.3/92.4 mmHg to 134.1/83.1 mmHg following treatment initiation. Only 26.7% of patients achieved the target BP (<130/<80 mmHg). This level is the universal target in the Japanese Society of Hypertension Guidelines for the Management of Elevated Blood Pressure and Hypertension 2025, highlighting a significant public health challenge. Unadjusted BP control rates varied by 10.2% across prefectures, narrowing to 7.4% after adjusting for individual-level patient characteristics. Pre-treatment SBP was the strongest predictor of post-treatment BP control. Ecological analysis revealed that each 1% increase in patients achieving the target BP of <130/<80 mmHg was associated with 3.5 fewer cerebrovascular disease deaths per 100,000 population in both sexes. PUDI showed a significant positive association with BP control rate (weighted Pearson’s *r* = 0.47; *p* < 0.001). In conclusion, substantial regional disparities in BP control persist across Japan, which are significantly influenced by physician availability and associated with differences in stroke mortality.

**Figure:**
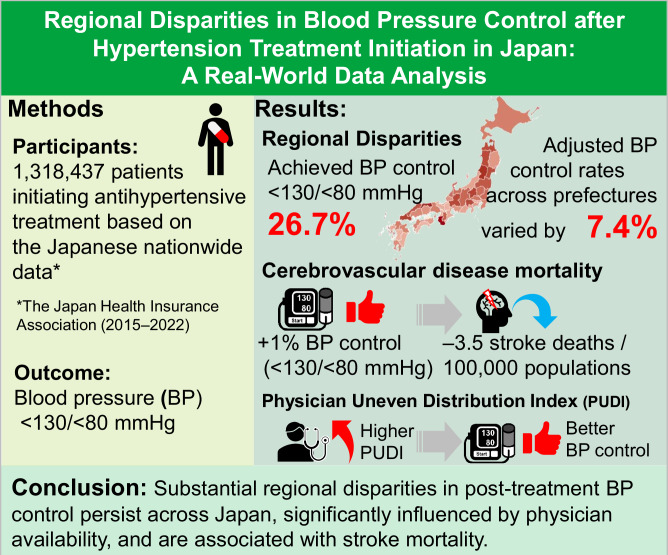
Using Japanese nationwide health check-up data from 1.3 million individuals initiating antihypertensive treatment, only 26.7% achieved the target BP (<130/<80 mmHg). Adjusted BP control rates varied by 7.4% across prefectures and were positively associated with physician availability and inversely with stroke mortality, indicating the impact of regional healthcare resource disparities.

Using Japanese nationwide health check-up data from 1.3 million individuals initiating antihypertensive treatment, only 26.7% achieved the target BP (<130/<80 mmHg). Adjusted BP control rates varied by 7.4% across prefectures and were positively associated with physician availability and inversely with stroke mortality, indicating the impact of regional healthcare resource disparities.

## Introduction

Hypertension is a major risk factor for cardiovascular diseases including myocardial infarction and stroke, affecting an estimated 43 million individuals in Japan [1]. Despite the substantial therapeutic advances that have enabled blood pressure (BP) control in most patients, BP control remains insufficient [2, 3]. This treatment gap, which is often referred to as the “hypertension paradox,” is largely attributable to clinical inertia, whereby healthcare providers fail to intensify treatment even when BP remains above target thresholds [4]. Considering that hypertension is one of the most prominent modifiable risk factors for cardiovascular morbidity and mortality in Japan, addressing this treatment gap is essential [5].

Globally, both geographic and socioeconomic disparities have been shown to significantly affect the prevalence and management [2, 6]. Geographic disparities in the quality and outcomes of hypertension management may exist across geographic regions within Japan, as suggested by official data showing regional variations in healthcare expenditures [7]. However, the regional disparities in the BP control rates remain inadequately characterized. As possible factors associated with regional disparities in BP control, pre-treatment characteristics, especially BP immediately before treatment initiation, are critical determinants of subsequent BP control [8]. Furthermore, regional differences in BP control may be directly influenced by healthcare-system-related factors. Understanding these disparities in the Japanese context, where healthcare access barriers are reduced relative to other countries’ healthcare systems, may reveal factors contributing to uncontrolled BP beyond traditional medical access issues [3]. The recent release of the Japanese Society of Hypertension Guidelines for the Management of Elevated Blood Pressure and Hypertension 2025 (JSH2025), recommending a universal target BP of <130/<80 mmHg regardless of age, makes a detailed understanding of these real-world disparities particularly urgent [9].

This study primarily aimed to assess the regional disparities in BP control rates after the initiation of antihypertensive therapy and to explore its associations with regional healthcare resources across 47 prefectures in Japan. We evaluated BP control using nationwide health check-up data from the Japan Health Insurance Association (JHIA), which includes both employer-based and lifestyle disease prevention health check-ups. This study also sought to assess longitudinal changes in BP before and after the initiation of antihypertensive therapy. Consideration of the pre-treatment status allows us to incorporate pre-treatment measurements and baseline characteristics immediately before treatment initiation. It also minimizes confounding from accumulated treatment exposure, including the treatment duration.

## Methods

### Study design and data source

This retrospective cohort study utilized health check-up data collected between the fiscal years 2015 and 2022 (April 1, 2015, to March 31, 2023) from the administrative database maintained by the JHIA (Kyokai Kenpo), the largest government-accredited public health insurer in Japan. The JHIA provides coverage to ~40 million individuals, including 25 million insured employees and 15 million dependent family members, primarily representing employees of small- to medium-sized enterprises and their dependents [10]. Under national mandates, all public health insurers in Japan, including the JHIA, are required to offer annual health screenings to members aged 40–74 years.

### Participant selection

**Fig. 1:**
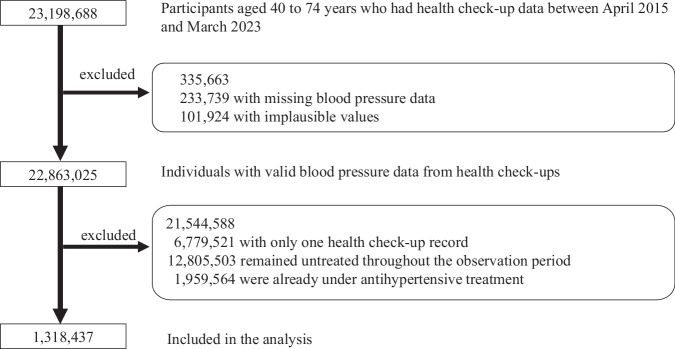
Flow chart of patient selection

Figure 1 presents a flowchart of the patient selection process. Overall, 23,198,688 individuals with valid health check-up data were identified from the national database of employer-based health check-ups and health check-ups for the prevention of lifestyle-related diseases, both administered by the JHIA between fiscal years 2015 and 2022. Individuals without valid BP measurements at either the pre- or post-treatment health check-ups were excluded, as these values were essential for outcome assessment. Subsequently, physiologically implausible values, such as cases in which DBP exceeded SBP, were removed, as they were considered likely data entry errors. From the remaining 22,863,025 individuals, we excluded those with only one health check-up record, those who remained untreated throughout the observation period, and those who were already under antihypertensive treatment. Among them, 1,318,437 who initiated antihypertensive treatment were included in the present analysis.

### Data collection

The following variables were extracted from the health check-up data: systolic and diastolic BP (SBP and DBP), body mass index (BMI), hemoglobin A1c (HbA1c), low-density lipoprotein cholesterol (LDL-C), high-density lipoprotein cholesterol (HDL-C), and estimated glomerular filtration rate (eGFR), which was calculated using a creatinine formula modified for Japanese individuals: eGFR = 194 × serum creatinine^−1.094^ × age^−0.287^ × *α* (*α* = 0.739 for women and *α* = 1 for men) [11]. Data on smoking, alcohol consumption, treatment, and medical history were obtained from a standard questionnaire used in the health check-up. Data on corporate type, industry classification, and annual income were obtained from the records maintained by the JHIA. Corporate entity type was categorized as either corporation-employed or self-employed. Industry classification was grouped into primary (e.g., agriculture, forestry, and fisheries), secondary (e.g., mining and quarrying, construction, and manufacturing), and tertiary sectors based on standard industrial classifications in Japan. Although standard measurement protocols such as averaging multiple readings after a period of rest are recommended for BP assessment, actual measurement conditions may vary across institutions performing health check-ups [12]. Smoking and alcohol use were defined as “present” if reported as current at the time of the health check-up.

### Antihypertensive treatment initiation and BP categories

Use of antihypertensive medication was defined based on responses to the self-administered health check-up questionnaire, specifically the item, “Are you currently taking medication to lower your BP?” Individuals who responded “yes” to this question for the first time following a prior “no” response were identified as newly initiating antihypertensive treatment.

We used BP classification following the Japanese Society of Hypertension Guidelines for the Management of Hypertension 2019 (JSH 2019), with SBP or DBP (whichever was higher) to assign individuals to one of six categories: SBP/DBP < 120/<80 mmHg, SBP/DBP 120–129/<80 mmHg, SBP/DBP 130–139/80–89 mmHg, SBP/DBP 140–159/90–99 mmHg, SBP/DBP 160–179/100–109 mmHg, and SBP/DBP ≥ 180/≥110 mmHg [1]. Additionally, we set two BP control targets during the treatment phase: SBP/DBP < 130/<80 mmHg and <140/<90 mmHg during the treatment phase [1].

### Variables used in ecological analyses

Owing to the strong association between BP and stroke in Asian populations, we focused on cerebrovascular disease mortality as a key health outcome [13]. We obtained sex-specific, age-adjusted mortality rates for each prefecture from publicly available official vital statistics of Japan compiled by the Ministry of Health, Labour and Welfare [14].

To investigate regional factors associated with variations in BP control, we collected the six indicators relevant to BP: (1) the Physician Uneven Distribution Index (PUDI) (published January 2024) [15], (2) the number of outpatient claims for ambulatory BP monitoring (ABPM) per 100,000 population (9th NDB Open Data) [16], (3) the average daily number of outpatients per 100,000 population (2020 Survey of Medical Institutions and Hospital Report) [17], (4) the health insurance premium rate in the 2022 fiscal year [18], (5) the participation rate in specific health check-ups in the 2022 fiscal year [19], and (6) the number of general hospital beds per 100,000 population (2023 Survey of Medical Institutions and Hospital Report) [20]. These data were collected from publicly available data in Japan.

The PUDI is a nationally standardized policy index developed by Japan’s Ministry of Health, Labour, and Welfare, compares medical supply (e.g., physicians’ age- and sex-adjusted working hours) with estimated medical demand, including age-adjusted consultation rates and population flows, to more accurately evaluate physician distribution [21]. A higher PUDI value indicates a greater supply of physicians relative to regional demand. The Hospital Pharmacist Maldistribution Index used in this study is an analogous indicator developed based on the same conceptual framework as the PUDI, which compares pharmacist supply (adjusted working hours) with estimated demand (workload) (June 2023) [22]. A detailed description of the PUDI, including its calculation formula, has been previously published [23].

### Statistical analysis

The magnitude of change in characteristics following antihypertensive treatment initiation was assessed using standardized mean differences (SMD), with absolute values ≥ 0.1 indicating clinically meaningful differences. To identify the factors associated with post-treatment SBP, multiple linear regression analysis was performed with post-treatment characteristics, pre-treatment SBP values, months of post-treatment check-ups, and a binary indicator for the period relative to the 2019 JSH guideline publication, released on April 25, 2019 (0 = pre-publication, 1 = post-publication) as independent variables. The relative importance of each predictor was evaluated using the partial coefficient of determination (Partial *R*²).

To evaluate regional variations in BP control, the mean SBP values and proportion of individuals achieving the target BP control (<130/<80 mmHg and <140/<90 mmHg) were calculated for each prefecture. Adjusted means and proportions were estimated using the analysis of covariance and robust Poisson regression models, respectively. The adjusted models included the following covariates; sex, the post-treatment health check-up characteristics including age, BMI, LDL-C, HDL-C, HbA1c ≥ 6.5%, eGFR < 60 mL/min/1.73 m², proteinuria, history of stroke, cardiovascular disease, or kidney disease, smoking status, alcohol consumption status, anti-diabetic treatment, lipid-lowering treatment, and recent corporate entity type, recent industry classification, interval between health check-ups, the month of the post-treatment health check-ups, annual income, and the period before or after the 2019 JSH guideline publication, pre-treatment variables included SBP values and the month of the pre-treatment check-up. For models evaluating regional variation, Tokyo, a Japanese metropolis, was used as the reference prefecture.

We conducted a prefecture-level ecological study using all 47 prefectures in Japan as units of analysis. The association between the prefecture-level-adjusted BP control rate and age-adjusted cerebrovascular disease mortality rate was analyzed using Pearson’s correlation and simple linear regression analysis. Sex-specific analyses were conducted as mortality data from the 2020 Vital Statistics of Japan were reported separately for men and women. Furthermore, to explore the potential determinants of regional variation in BP control, we performed another ecological analysis using multiple linear regression to assess the association between the adjusted BP control rate and the six healthcare resource indicators. All ecological data analyses were weighted by the number of individuals in each prefecture.

Missing data were handled as follows: For continuous variables with a low proportion of missing values, such as BMI (*n* = 58), LDL-C (*n* = 1095), and HDL-C (*n* = 662), data were imputed using a single imputation from a sex-stratified linear regression model, with age as the predictor. For variables with a substantial proportion of missing data, including disease history and proteinuria, the missing values were treated as separate categories and included as indicator variables in the statistical models to account for potential bias. To assess robustness, we conducted a sensitivity analysis using multiple imputations by chained equations (MICE). Given computational constraints in the mandatory secure analysis environment, we implemented MICE with *m* = 20 as a pragmatic compromise. All statistical analyses were conducted using SAS software (version 9.4; SAS Institute Inc., Cary, NC, USA) and R software version 4.5.1 for map generation. Statistical significance was set at *p* < 0.05.

## Results

### Pre-treatment and post-treatment characteristics of individuals initiating antihypertensive treatment

The pre- and post-treatment characteristics are shown in Table 1. Post-treatment BP refers to the office BP measured at the health check-up when antihypertensive medication use was first reported. The interval between the pre- and post-treatment check-ups was tightly centered around 1 year (median: 1.0 year; interquartile range: 1.0–1.1 years; 5th–95th percentile: 0.8–2.0 years). At the pre-treatment health check-up, the mean age was 55.2 years, and 71.1% of the patients were male. The mean SBP/DBP decreased by 14.2 ± 19.6/9.3 ± 12.6 mmHg. Consequently, the proportion of individuals with SBP/DBP ≥ 140/90 mmHg declined markedly from 71.9% to 40.7%, but only 26.7% of the individuals achieved the target level of <130 mmHg/<80 mmHg at the post-treatment visit. Favorable changes were also observed in lipid profiles and glycemic control, accompanied by a substantial increase in the use of lipid-lowering agents (SMD = 0.46) and a significant reduction in LDL-C levels (SMD = –0.23). The proportion of patients achieving the target BP was similar before and after the 2019 JSH guideline revision (Supplementary Table 1).

**Table 1:** Pre-treatment and post-treatment characteristics of individuals initiating antihypertensive treatment

Characteristic	Pre-treatment	Post-Treatment	Paired SMD
Total participants	1,318,437	1,318,437	–
Age, years	55.2 (7.9)	56.5 (7.9)	0.2
Age ≥65 years, %	12.6	15.5	0.09
Men, %	71.1	71.1	–
Median annual income, 10^4^ yen	336	336	0.04
BMI, kg/m^2^	25.2 (4.3)	25.2 (4.3)	0.00
LDL-cholesterol, mg/dL	129.1 (34.5)	121.1 (31.3)	–0.23
HDL-cholesterol, mg/dL	61.0 (17.4)	60.9 (17.4)	–0.01
HbA1c, %	5.9 (1.0)	5.8 (0.8)	–0.1
Missing data	816,155	800,533	–
eGFR, mL/min/1.73 m²	75.7 (15.4)	74.7 (15.7)	–0.06
Missing data	600,259	418,832	–
Current smoking, %	34.5	31.7	−0.06
Current drinking, %	38.0	36.5	−0.03
Receiving antihypertensive treatment, %	0.0	100.0	–
Diabetes, %	9.6	14.1	0.15
Receiving antidiabetic treatment, %	6.6	12.2	0.17
Dyslipidemia, %	60.0	61.3	0.03
Receiving lipid-lowering treatment, %	9.5	22.9	0.46
History of stroke, %	1.7	3.7	0.1
Missing data	185,451	153,087	–
History of cardiovascular disease, %	4.3	7.8	0.2
Missing data	184,766	152,634	–
History of kidney disease, %	0.7	1.2	0.05
Missing data	186,718	154,130	–
Proteinuria, %	8.3	6.1	–0.08
Missing data	164,026	402,080	–
Interval between health check-ups, years	–	1.1 (0.5)	–
Median [interquartile range], years	–	1.0 (1.0–1.1)	–
5th–95th percentile, years	–	0.8–2.0	–
Corporate entity type: Corporation, %	98.9	98.9	–
Corporate entity type: Self-employed, %	1.1	1.1	–
Industry: Primary, %	0.9	0.9	–
Industry: Secondary, %	12.2	12.2	–
Industry: Tertiary, %	86.9	86.9	–
SBP, mm Hg	148.3 (20.7)	134.1 (16.8)	–0.69
DBP, mm Hg	92.4 (14.0)	83.1 (11.4)	–0.66
SBP/DBP < 120/<80 mmHg, %	5.7	14.8	0.4
SBP/DBP 120–129/<80 mmHg, %	5.2	11.9	0.31
SBP/DBP 130–139/ 80–89 mmHg, %	17.3	32.6	0.41
SBP/DBP 140–159/ 90–99 mmHg, %	31.7	29.7	–0.04
SBP/DBP 160–179/ 100–109 mmHg, %	26.9	8.9	–0.41
SBP/DBP ≥ 180/≥110 mmHg, %	13.3	2.1	–0.33

Data are presented as means (standard deviation), medians, or percentages. All calculations were based on non-missing values

*SMD* standardized mean difference, *MI* body mass index, *DBP* diastolic blood pressure, *eGFR* estimated glomerular filtration rate, *HbA1c* hemoglobin A1c, *HDL* high-density lipoprotein, *LDL* low-density lipoprotein, *SBP* systolic blood pressure

In the multiple linear regression analysis, pre-treatment SBP was the strongest predictor of the post-treatment SBP (Partial *R*² = 19.61%, Pearson’s correlation coefficient = 0.47), followed by BMI (Partial *R*² =  1.00%), age (Partial *R*² = 0.50%), HDL cholesterol (Partial *R*² = 0.49%), proteinuria negative (Partial *R*² = 0.20%), annual income (Partial *R*² =  0.19%), male sex (Partial *R*² = 0.18%), LDL cholesterol (Partial *R*² =  0.18%), and eGFR ≥ 60 mL/min/1.73 m² (Partial *R*² = 0.18%), with all other factors showing partial *R*² values < 0.10% (Supplementary Table 2).

### Regional differences in SBP before and after initiation of antihypertensive treatment

Prior to the initiation of antihypertensive treatment, the mean SBP differed by up to 8.3 mmHg across prefectures, ranging from 153.1 mmHg in the Tottori Prefecture to 144.8 mmHg in the Tokushima Prefecture (Supplementary Fig. 1). The geographical distribution is shown in Supplementary Fig. 2.

**Fig. 2:**
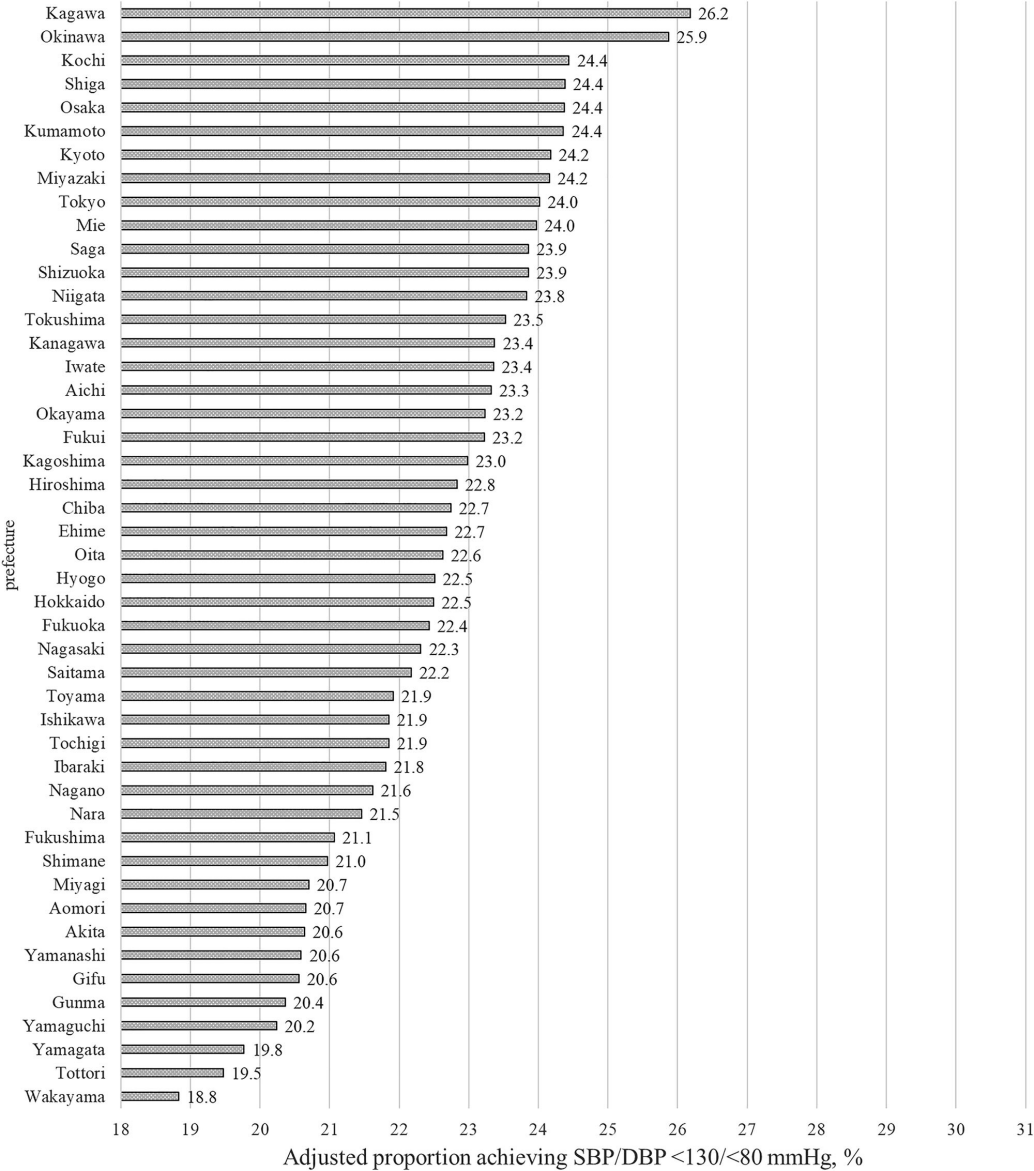
Adjusted proportion of individuals with post-treatment systolic/diastolic BP < 130/<80 mmHg (%). Results are adjusted for sex, corporate entity type, and industry classification. Variables measured at the post-treatment health check-up included age, body mass index, low-density lipoprotein cholesterol, smoking status, alcohol consumption status, antidiabetic treatment, interval between health check-ups, month of the post-treatment health check-up, annual income, and a binary indicator for the JSH2019 guideline period (pre/post-publication). Furthermore, pre-treatment variables included systolic BP and the month of the pre-treatment check-up. Models for evaluating regional variation. BP blood pressure

The unadjusted proportion of patients with BP < 130/<80 mmHg differed by a maximum of 10.2% across the 47 prefectures in Japan, ranging from 20.3% in Tottori to 30.5% in Okinawa (Supplementary Fig. 3). Adjusting for pre-treatment characteristics narrowed the observed regional disparities and changed the prefectural rankings; the adjusted proportion of patients with BP controlled to <130/<80 mmHg differed by a maximum of 7.4%, from a low of 18.8% in Wakayama to a high of 26.2% in Kagawa (Fig. 2). Results from the multiple-imputation sensitivity analysis were consistent with those of the primary analysis, corroborating robustness (Supplementary Fig. 4).

The proportion of patients with BP controlled to <140/<90 mmHg differed by a maximum of 13.1% before adjusting for patient characteristics, from 51.0% in Tottori to 64.1% in Saga (Supplementary Fig. 5), while the maximum regional difference narrowed to 9.5% (Supplementary Fig. 6). The unadjusted mean post-treatment SBP differed by a maximum of 5.3 mmHg before adjustments (Supplementary Fig. 7) while it was narrowed to 3.2 mmHg after adjustments (Supplementary Fig. 8). Sex stratified results are shown in Supplementary Figs. 9–11. The adjusted proportion achieving BP < 130/<80 mmHg ranged from 17.1% to 24.5% for men (7.4% difference) and 23.1% to 31.0% for women (7.9% difference) across prefectures (Supplementary Fig. 9).

### Prefecture-level BP control with age-adjusted stroke mortality

**Fig. 3:**
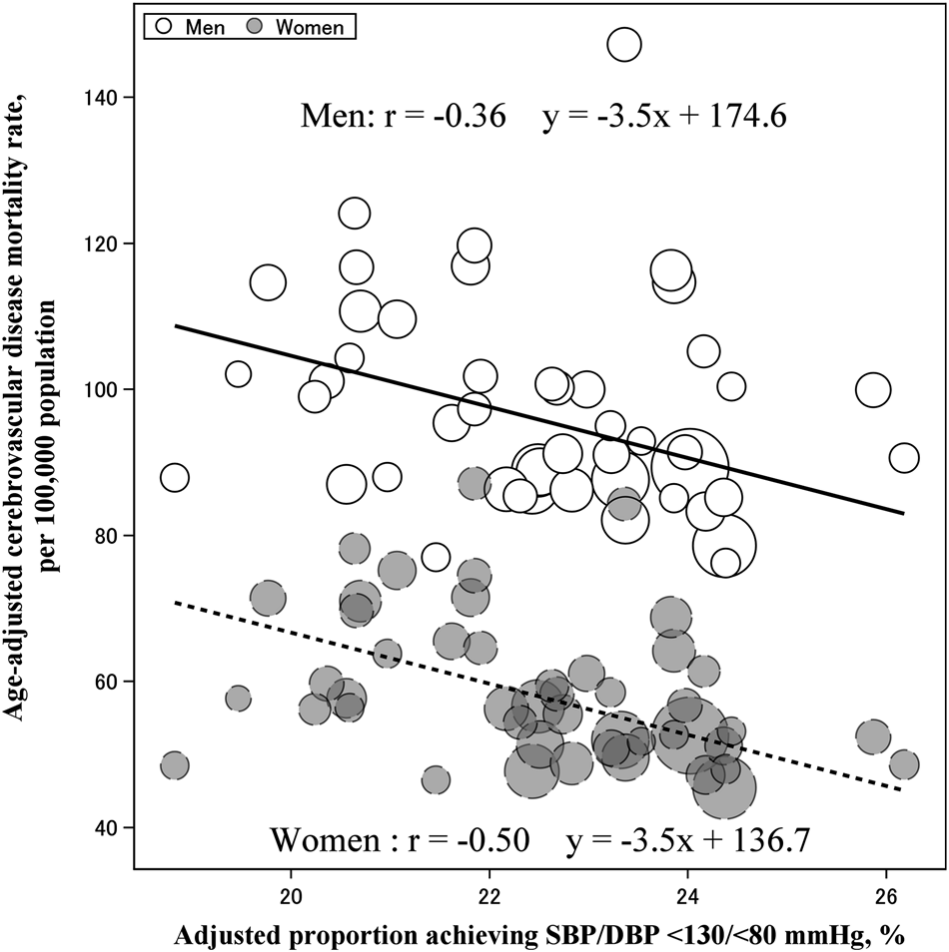
Scatter plot between the adjusted proportion of individuals achieving systolic/diastolic BP < 130/<80 mmHg and the age-adjusted cerebrovascular disease mortality rate across prefectures. Prefecture-level BP control is driven by variables in Fig. 2. Prefecture-level BP control values are the same as those shown in Fig. 2. Age-adjusted cerebrovascular disease mortality rates (per 100,000 people) were obtained from publicly available statistics in Japan. BP blood pressure

In the prefecture-level ecological analysis, a higher adjusted proportion of patients with BP < 130/<80 mmHg was associated with a lower age-adjusted cerebrovascular disease mortality rate in both men and women (Fig. 3). Weighted linear regression analysis suggested that the age-adjusted mortality rate decreased by 3.5 per 100,000 people for men and 3.5 per 100,000 people for women for every 1% increase in patients with controlled BP < 130/<80 mmHg.

### Associated with prefecture-level BP control

**Fig. 4:**
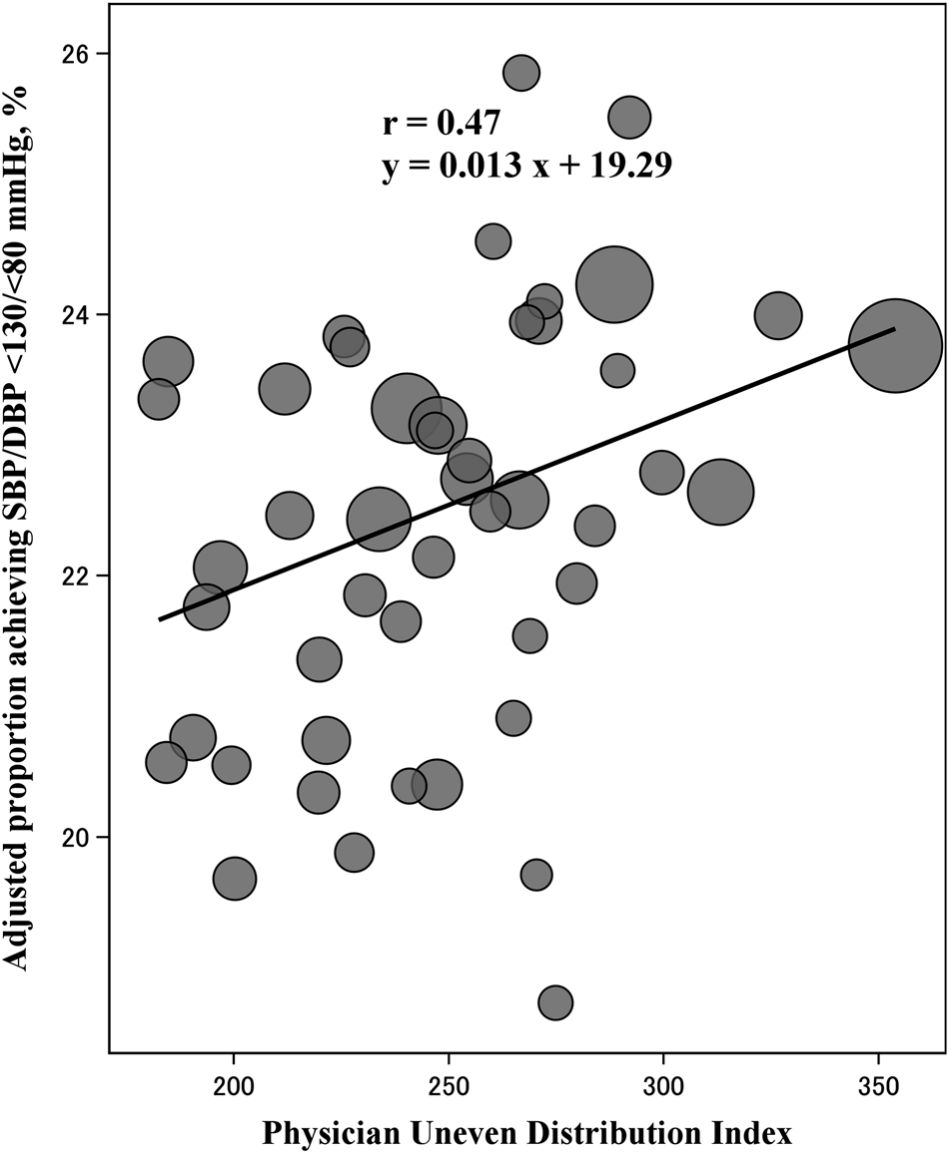
Scatter plot between the adjusted proportion of individuals with systolic/diastolic BP < 130/<80 mmHg and the PUDI across prefectures. Prefecture-level BP control values are the same as those shown in Fig. 2. PUDI (number of physicians per 100,000 people) was obtained from publicly available statistics in Japan. BP blood pressure, PUDI Physician Uneven Distribution Index

The model examined the independent associations between the six healthcare resource indicators and the prefecture-level adjusted BP control rate (Table 2). In the multivariate model, PUDI was the only factor significantly associated with the proportion of patients with BP controlled to <130/<80 mmHg, whereas the number of ABPM claims, Average Daily Number of Outpatients, Health Insurance Premium Rate, specific health check-up participation rate, and hospital bed availability were not significantly associated with BP control. Prefectures with a higher PUDI had a higher proportion of patients with BP < 130/<80 mmHg (weighted Pearson’s *r* = 0.47; *p* < 0.001) (Fig. 4). When the Hospital Pharmacist Maldistribution Index was used instead of PUDI, a similar significant association was observed (Supplementary Table 3). A positive correlation was also observed in the ecological analysis (weighted Pearson’s *r* = 0.50; *p* < 0.001) (Supplementary Fig. 12).

**Table 2:** Multiple linear regression analysis of the association between healthcare resource indicators and prefecture-level BP control rates

Prefecture-level indicator	*B*	SE	*t*-value	*P*-value
PUDI, per 1-point increase	0.023	0.007	3.41	0.0015
Outpatient ABPM claims, per 1 claim per 100,000 population	0.019	0.014	1.30	0.20
Average daily number of outpatients, per 1 person per 100,000 population	0.003	0.003	1.06	0.30
Health insurance premium rate, per 1% increase	–0.673	0.885	–0.76	0.45
Participation rate in specific health check-ups, per 1% increase	–0.105	0.055	–1.92	0.062
Number of general hospital beds, per 1 bed per 100,000 population	–0.006	0.003	–1.72	0.094

Results of the weighted multiple linear regression analysis. Dependent variable was the prefecture-level-adjusted proportion of individuals achieving a systolic/diastolic blood pressure of <130/<80 mmHg by prefecture (Fig. 2). All six indicators were simultaneously entered into the model. The analysis was weighted according to the number of participants in each prefecture

*B* unstandardized regression coefficient, *SE* standard error, *PUDI* Physician Uneven Distribution Index, *ABPM* ambulatory blood pressure monitoring

## Discussion

This study revealed substantial regional disparities in BP control across 47 prefectures in Japan even after accounting for patient characteristics. While antihypertensive treatment reduced the mean SBP/DBP by 14.2/9.3 mmHg, only 26.7% of patients achieved a BP target of <130/<80 mmHg. Regional disparities in BP control to <130/<80 mmHg reached a maximum of 7.4% across prefectures, even after adjusting for patient characteristics. Higher prefecture-level BP control was associated with reduced stroke mortality and was significantly influenced by PUDI, indicating physician availability per population.

Despite the initiation of antihypertensive therapy, most patients did not achieve a BP target of <130/<80 mmHg, with 73.3% not reaching this target post-treatment. Previous studies have shown low hypertension control rates in Japan, although they generally used a standard of <140/90 mmHg [24, 25]. Therefore, it is expected that the results would be considerably lower when the current target of <130/<80 mmHg is used. A recent multicenter survey from Kanagawa Prefecture in Japan reported that 68.7% and 77.2% patients did not achieve office BP < 130/<80 mmHg and home BP < 125/<75 mmHg, respectively, in 2022 [26]. This result may indicate worse BP control in home measurement than in office measurement. Meanwhile, management by hypertension specialists can improve target BP achievement rates [27]. A sub-analysis of the Ohasama study demonstrated excellent home BP control under hypertension-specialized medical care [28]. Therefore, measures for improving BP control under non-specialized medical care should be considered.

High pre-treatment BP was a major contributor to poor BP control after treatment initiation, consistent with the findings of previous studies in different Japanese populations [3, 29]. Theoretically, good BP control should be achieved if treatment is appropriately intensified, regardless of pre-treatment BP levels; thus, there is a weak association between pre- and post-treatment BP levels. The strong association between pre-treatment BP levels and uncontrolled BP suggests inadequate intensification of antihypertensive treatment, a pattern consistent with clinical inertia [3]. The Hypertension Objective Treatment Based on Measurement by Electrical Devices of Blood Pressure (HOMED-BP) study used a centralized, algorithm-driven intensification protocol based on home BP measurements [30]. In that study, ~75% patients achieved home BP < 135/85 mmHg and the correlation between baseline and on-treatment home systolic BP was relatively weak (*r* = 0.24–0.25). In real-world settings, physicians may refrain from intensifying antihypertensive medication because of reluctance to adjust therapy, limited consultation time, and concerns about side effects [31]. These barriers likely contribute to clinical inertia. The strong association between pre-treatment BP levels and uncontrolled BP suggests inadequate intensification of antihypertensive treatment, a pattern consistent with clinical inertia [3]. Treatment delays for hypertension are associated with uncontrolled BP [32] and may result in a high risk of cardiovascular disease [33]. These findings underscore the importance of both timely initiation and sufficient intensification of antihypertensive therapy to achieve optimal BP control.

A key finding of this study was the significant interprefectural variation in BP control after treatment initiation, even after adjusting for pre-treatment SBP and other patient characteristics. Reducing these regional disparities is crucial for reducing the cardiovascular disease burden, because ecological analysis revealed a significant association between adjusted regional disparities in BP control and stroke mortality. Based on the observed association, the remaining 7.4% regional disparity in adjusted BP control to <130/<80 mmHg could translate to a difference of 25.9 deaths per 100,000 population in age-adjusted cerebrovascular disease mortality for both men and women between the highest- and lowest-performing prefectures. Overall, BP control rates were consistently lower in men.

Several possible factors contribute to the observed regional disparities in BP control. A large global analysis reported that hypertension care varies substantially worldwide and even within the same regions in the world [2]. It appears that high income countries have relatively better BP control rates than do low- and middle-income countries. Meanwhile, the present study showed geographic heterogeneity within a single country having universal health coverage, i.e., Japan. Previous large-scale studies based on claims data indicated regional disparities in hypertension prevalence [34] and the association between income and hypertension prevalence in Japan [35]. However, the present study demonstrated, for the first time to our knowledge, regional disparity in hypertension control in Japan after consideration of the socioeconomic status and pre-treatment BP. Furthermore, our study provides new evidence that these subnational disparities are associated with the availability of healthcare resources, particularly physician distribution.

At the subnational level, several possible factors contribute to the observed regional disparities in BP control. First, therapeutic delay, as suggested by high pre-treatment BP, may have contributed to the uncontrolled BP. A high pre-treatment BP was the strongest predictor of uncontrolled BP in the present study. Although regional differences in BP control were statistically adjusted for pre-treatment BP, residual confounding due to therapeutic delay may have remained. Secondly, regional variations in treatment intensification and overall clinical practice may have occurred. Our previous findings indicated that inadequate antihypertensive combination therapy was the primary contributor to antihypertensive treatment failure, which is considered as clinical inertia [3]. Third, shortages of physicians and co-medical staff may have contributed to regional disparities, as supported by another ecological analysis using the PUDI and Hospital Pharmacist Maldistribution Index. A larger number of physicians or comedicals may improve patient access, shorten waiting times, and allow more frequent follow-up visits, which may facilitate timely BP reassessment and dosage adjustment. The region-specific implementation of team-based care models may be a promising strategy for addressing this issue. Recent meta-analyses have revealed that team-based care [36] and interventions led by multiple healthcare professionals, especially those involving pharmacists and community health workers [37], resulted in greater SBP reduction. These possibilities suggest that regional disparities in BP control result from multifactorial causes including therapeutic delays, variations in clinical practice, and healthcare workforce distribution. Public health policies targeting these underlying factors may be effective in further reducing the cardiovascular disease burden at the population level.

This study had some limitations. First, the study population was limited to beneficiaries of the JHIA, primarily covering employees of small- and medium-sized enterprises in Japan, which may limit the generalizability and transportability to the broader population. Second, unmeasured confounders, such as diet, physical activity, medication adherence, and other socioeconomic factors, may have influenced the results. Furthermore, because we did not use claims data in the present study, information on actual antihypertensive medications was lacking. Although the multivariable model included the interval between pre- and post-treatment health check-ups as an adjustment factor, the duration between the actual time of antihypertensive treatment initiation and the post-treatment BP measurement could have been a confounding factor for the regional disparity in BP control. Meanwhile, our previous real-world analysis showed that BP reduction within the first treatment year reached >90% of the BP reduction plateau achieved in subsequent years [38]. Third, patients included in the analyses were those who had initiated antihypertensive treatment. Exclusion of patients who were already receiving antihypertensive therapy may have limited the comprehensiveness of the findings, and our estimates may not fully generalize to patients under long-term treatment. There could have been regional disparities in the proportions of patients who did not attend health check-ups or visit medical institutions, and those who did not initiate treatments. These disparities may further explain the differences in cardiovascular diseases among the regions. Fourth, although the regional disparity in BP control was almost similar among men and women, the sex-stratified analyses suggested that sex differences may have influenced the results. Future studies should explore the factors that contribute to sex differences in regional BP control disparities. Finally, our ecological analyses were subject to ecological fallacy. The prefecture-level association observed between PUDI and BP control rates does not necessarily imply a causal relationship at the individual level.

In conclusion, this nationwide, real-world analysis revealed persistent regional disparities in BP control across Japan, even after adjusting for patient-level factors. After treatment initiation, the proportion of patients achieving BP < 130/<80 mmHg was only 26.7%, with a maximum difference of 7.4% between the regions. This achievement rate is remarkably low and highlights a significant public health challenge, especially considering that the recent JSH 2025 recommend a universal target BP of <130/<80 mmHg regardless of age. These disparities were significantly associated with stroke mortality and physician availability, suggesting that the healthcare resource distribution affects population-level treatment outcomes. Regional disparities in BP control may stem from differences in clinical inertia and physician availability. National policies that promote the equitable distribution of multidisciplinary healthcare professionals may reduce the burden of cardiovascular disease by improving hypertension control.

## Supplementary information

Supplementary Material

## References

[1] Umemura S, Arima H, Arima S, Asayama K, Dohi Y, Hirooka Y, et al. The Japanese Society of Hypertension Guidelines for the management of hypertension (JSH 2019). Hypertens Res. 2019;42:1235–481. 31375757 10.1038/s41440-019-0284-9

[2] NCD Risk Factor Collaboration. Worldwide trends in hypertension prevalence and progress in treatment and control from 1990 to 2019: a pooled analysis of 1201 population-representative studies with 104 million participants. Lancet. 2021;398:957–80. 34450083 10.1016/S0140-6736(21)01330-1 PMC8446938

[3] Satoh M, Muroya T, Murakami T, Obara T, Asayama K, Ohkubo T, et al. The impact of clinical inertia on uncontrolled blood pressure in treated hypertension: real-world, longitudinal data from Japan. Hypertens Res. 2024;47:598–607. 37872377 10.1038/s41440-023-01452-2

[4] Phillips LS, Branch WT, Cook CB, Doyle JP, El-Kebbi IM, Gallina DL, et al. Clinical inertia. Ann Intern Med. 2001;135:825–34. 11694107 10.7326/0003-4819-135-9-200111060-00012

[5] Liu L, Miura K, Fujiyoshi A, Kadota A, Miyagawa N, Nakamura Y, et al. Impact of metabolic syndrome on the risk of cardiovascular disease mortality in the United States and in Japan. Am J Cardiol. 2014;113:84–9. 24169008 10.1016/j.amjcard.2013.08.042

[6] Chaturvedi A, Zhu A, Gadela NV, Prabhakaran D, Jafar TH. Social determinants of health and disparities in hypertension and cardiovascular diseases. Hypertension. 2024;81:387–99. 38152897 10.1161/HYPERTENSIONAHA.123.21354 PMC10863660

[7] Investigation Division HIB, Ministry of Health, Labour and Welfare. Regional disparity analysis of medical expenses for fiscal year 2022 (computer-processed data). Tokyo: Ministry of Health, Labour and Welfare; 2024.

[8] Sano H, Hara A, Asayama K, Miyazaki S, Kikuya M, Imai Y, et al. Antihypertensive drug effects according to the pretreatment self-measured home blood pressure: the HOMED-BP study. BMJ Open. 2020;10:e040524. 33310801 10.1136/bmjopen-2020-040524 PMC7735093

[9] Ohya Y, Sakima A, Arima H, Fukami A, Furuhashi M, Ishida M, et al. Key highlights of the Japanese Society of Hypertension Guidelines for the management of elevated blood pressure and hypertension 2025 (JSH2025). Hypertens Res. 2025. 10.1038/s41440-025-02331-8). 10.1038/s41440-025-02331-8 40877471

[10] Yagi R, Mori Y, Goto S, Iwami T, Inoue K. Routine electrocardiogram screening and cardiovascular disease events in adults. JAMA Intern Med. 2024;184:1035–44. 38949831 10.1001/jamainternmed.2024.2270 PMC11217891

[11] Matsuo S, Imai E, Horio M, Yasuda Y, Tomita K, Nitta K, et al. Revised equations for estimated GFR from serum creatinine in Japan. Am J Kidney Dis. 2009;53:982–92. 19339088 10.1053/j.ajkd.2008.12.034

[12] Satoh M, Nakayama S, Toyama M, Hashimoto H, Murakami T, Metoki H. Usefulness and caveats of real-world data for research on hypertension and its association with cardiovascular or renal disease in Japan. Hypertens Res. 2024;47:3099–113. 39261703 10.1038/s41440-024-01875-5 PMC11534704

[13] Lawes CM, Rodgers A, Bennett DA, Parag V, Suh I, Ueshima H, et al. Blood pressure and cardiovascular disease in the Asia Pacific region. J Hypertens. 2003;21:707–16. 12658016 10.1097/00004872-200304000-00013

[14] Statistics and Information Department MoH, Labour and Welfare. Overview of age-adjusted mortality rates by prefecture in 2020. Tokyo: Ministry of Health, Labour and Welfare; 2023.

[15] Ministry of Health Law. Physician uneven distribution index. Tokyo: Ministry of Health, Labour and Welfare; 2024.

[16] Ministry of Health Law. The 9th NDB Open Data: Number of claims for Section D (Laboratory tests) by prefecture (FY2022). In: Ministry of Health Law. https://www.mhlw.go.jp/content/12400000/001258315.xlsx. Tokyo, 2024.

[17] Ministry of Health Law. Summary of the 2020 Survey of Medical Institutions (Static/Dynamic) (Final Figures) and Hospital Report. Tokyo: Ministry of Health, Labour and Welfare; 2022.

[18] Association JHI. The Japan Health Insurance Association’s premium rates for fiscal year 2022 will be revised from the March portion (paid in April). https://www.kyoukaikenpo.or.jp/g7/cat330/sb3130/r4/220202/.

[19] Ministry of Health LaW. Implementation Status of Specific Health Check-ups (FY2022): Number of eligible persons, participants, and participation rate. In: Ministry of Health LaW. https://www.mhlw.go.jp/content/12400000/001250945.xlsx. Tokyo, 2024.

[20] Ministry of Health LaW. Summary of the 2023 Survey of Medical Institutions (Static/Dynamic) and Hospital Report. Ministry of Health, Labour and Welfare: Tokyo; November 22 2024.

[21] Takayama A, Poudyal H. Assessing the Association of Physician and Specialist Maldistribution with out-of-hospital cardiac arrest outcomes: implications for regulatory policy. JMA J. 2025;8:506–16. 40416042 10.31662/jmaj.2024-0241 PMC12095126

[22] Ministry of Health Law. Regarding pharmacist supply and demand trends and measures to address uneven distribution. Tokyo: Ministry of Health, Labour and Welfare; 2023.

[23] Takayama A, Poudyal H. Incorporating medical supply and demand into the index of physician maldistribution improves the sensitivity to healthcare outcomes. J Clin Med. 2021;11:155. 10.3390/jcm11010155 PMC8745359 35011896

[24] Hisamatsu T, Segawa H, Kadota A, Ohkubo T, Arima H, Miura K. Epidemiology of hypertension in Japan: beyond the new 2019 Japanese guidelines. Hypertens Res. 2020;43:1344–51. 32636526 10.1038/s41440-020-0508-z

[25] NCD Risk Factor Collaboration. Long-term and recent trends in hypertension awareness, treatment, and control in 12 high-income countries: an analysis of 123 nationally representative surveys. Lancet. 2019;394:639–51. 31327564 10.1016/S0140-6736(19)31145-6 PMC6717084

[26] Kobayashi K, Chin K, Hatori N, Furuki T, Sakai H, Miyakawa M, et al. Cross-sectional survey of hypertension management in clinical practice in Japan: the Kanagawa Hypertension Study 2021 conducted in collaboration with Japan Medical Association Database of Clinical Medicine. Hypertens Res. 2023;46:2447–59. 37532949 10.1038/s41440-023-01366-z

[27] Sakima A, Yamazato M, Kohagura K, Ishida A, Matayoshi T, Tana T, et al. Achievement rate of target blood pressure in patients with hypertension treated by hypertension specialists and non-specialists in a real-world setting. Hypertens Res. 2023;46:2460–9. 37414873 10.1038/s41440-023-01362-3

[28] Satoh M, Metoki H, Murakami T, Tatsumi Y, Asayama K, Kikuya M, et al. Home blood pressure control and prescribing patterns of anti-hypertensive medications in a home blood pressure-based hypertension-specialized clinic in Japan: a sub-analysis of the Ohasama study. Hypertens Res. 2025;48:26–36. 39463432 10.1038/s41440-024-01954-7 PMC11700850

[29] Kitaoka K, Kaneko H, Suzuki Y, Okada A, Mizuno A, Fujiu K, et al. Blood pressure control and treatment status at 1 year after the first health check-up in individuals with observed referral-level blood pressure. Hypertens Res. 2025. 10.1038/s41440-025-02284-y. 10.1038/s41440-025-02284-y PMC12497642 40715802

[30] Asayama K, Ohkubo T, Metoki H, Obara T, Inoue R, Kikuya M, et al. Cardiovascular outcomes in the first trial of antihypertensive therapy guided by self-measured home blood pressure. Hypertens Res. 2012;35:1102–10. 22895063 10.1038/hr.2012.125

[31] Satoh M, Nobayashi H, Iwabe Y. Delayed treatment initiation as one of the crucial factors in clinical inertia: lessons from a real-world database research. Hypertens Res. 2025. 10.1038/s41440-025-02320-x. 10.1038/s41440-025-02320-x 40764677

[32] Barrett RB, Riesser B, Martin B, Sachdev N, Rakotz MK, Sutherland SE, et al. Treatment in the first month after hypertension diagnosis improves blood pressure control. Hypertension. 2025;82:1129–36. 40255193 10.1161/HYPERTENSIONAHA.124.23508 PMC12071505

[33] Ito H, Seki T, Kawazoe Y, Takiguchi T, Akagi Y, Kubota K, et al. Prognostic impact of the timing of antihypertensive medication initiation for hypertension detected at health screening on primary prevention of adverse cardiovascular events: Age-stratified real-world data analysis. Hypertens Res. 2025. 10.1038/s41440-025-02249-1. 10.1038/s41440-025-02249-1 PMC12411247 40537613

[34] Waki T, Miura K, Tanaka-Mizuno S, Ohya Y, Node K, Itoh H, et al. Prevalence of hypertensive diseases and treated hypertensive patients in Japan: a nationwide administrative claims database study. Hypertens Res. 2022;45:1123–33. 35681039 10.1038/s41440-022-00924-1

[35] Aida J, Inoue Y, Tabuchi T, Kondo N. Modifiable risk factors of inequalities in hypertension: analysis of 100 million health checkups recipients. Hypertens Res. 2024;47:1555–66. 38443615 10.1038/s41440-024-01615-9

[36] Akasaki Y, Suematsu Y, Azushima K, Shiga Y, Sakima A, Satoh M, et al. Impact of patient care teams on blood pressure control in patients with hypertension: a systematic review and meta-analysis. Hypertens Res. 2025. 10.1038/s41440-025-02152-9. 10.1038/s41440-025-02152-9 PMC12137128 39962166

[37] Mills KT, O’Connell SS, Pan M, Obst KM, He H, He J. Role of health care professionals in the success of blood pressure control interventions in patients with hypertension: a meta-analysis. Circ Cardiovasc Qual Outcomes. 2024;17:e010396. 39027934 10.1161/CIRCOUTCOMES.123.010396 PMC11338746

[38] Satoh M, Hirose T, Satoh H, Nakayama S, Obara T, Murakami T, et al. Actual impact of angiotensin II receptor blocker or calcium channel blocker monotherapy on renal function in real-world patients. J Hypertens. 2022;40:1564–76. 35792108 10.1097/HJH.0000000000003186

