# Supplementary material for: Regional disparities in blood pressure control after hypertension treatment initiation in Japan: a real-world data analysis
Source: Hypertens Res. 2025 Nov 18;49(2):328–39. doi: 10.1038/s41440-025-02454-y (PMC12823396; doi:10.1038/s41440-025-02454-y)
Supplement: Supplementary file 1 — Supplementary Material [file 41440_2025_2454_MOESM1_ESM.pdf]

Supplementary Material

The authors have provided this online data supplement to readers with additional information regarding this study.

Supplementary Table 1. Pre- and post-treatment characteristics of individuals initiating antihypertensive treatment, stratified according to the 2019 JSH guideline revision period

Supplementary Table 2. In the multiple linear regression model, systolic BP at the post-treatment health checkup was the dependent variable.

Supplementary Table 3. Multiple linear regression analysis of the association among healthcare resource indicators, Hospital Pharmacist Maldistribution Index, and prefecture-level BP control rates

Supplementary Figure 1. Unadjusted SBP (mmHg) at the pre-treatment health check-up by prefecture

Supplementary Figure 2. Geographic distribution of unadjusted SBP (mmHg) at the pre-treatment health check-up by prefecture

Supplementary Figure 3. Unadjusted proportion of individuals achieving SBP/DBP <130/<80 mmHg at the post-treatment health check-up (%) by prefecture

Supplementary Figure 4. Adjusted proportion of individuals achieving SBP/DBP <130/<80 mmHg at the post-treatment health check-up (%) by prefecture , estimated using multiple imputation.

Supplementary Figure 5. Unadjusted proportion of individuals achieving SBP/DBP <140/<90 mmHg at the post-treatment health check-up (%) by prefecture

Supplementary Figure 6. Adjusted proportion of individuals achieving SBP/DBP <140/<90 mmHg at the post-treatment health check-up (%) by prefecture

Supplementary Figure 7. Unadjusted SBP (mmHg) at the post-treatment health check-up by prefecture

Supplementary Figure 8. Adjusted SBP (mmHg) at the post-treatment health check-up by prefecture

Supplementary Figure 9. Unadjusted (left) and adjusted (right) proportion of men and women achieving SBP/DBP <130/<80 mmHg at the post-treatment health check-up (%) by prefecture

Supplementary Figure 10. Unadjusted (left) and adjusted (right) proportion of men and women achieving SBP/DBP <140/<90 mmHg at the post-treatment health check-up (%) by prefecture

Supplementary Figure 11. Unadjusted (left) and adjusted (right) SBP (mmHg) values for men and women at the post-treatment health check-up by prefecture

Supplementary Figure 12. Scatter plot between the adjusted proportion of individuals with systolic/diastolic BP <130/<80 mmHg and the Hospital Pharmacist Maldistribution Index across prefectures

Supplementary Table 1. Pre- and post-treatment characteristics of individuals initiating antihypertensive treatment, stratified according to the 2019 JSH guideline revision period

	before guideline revision			after guideline revision		
Characteristic	Pre treatment	Post-Treatment	Paired SMD	Pre treatment	Post-Treatment	Paired SMD
Total participants	482,485	482,485	-	835,952	835,952	-
Age, years	53.9 (8.6)	54.9 (8.6)	0.12	54.1 (8.7)	55.3 (8.7)	0.14
Age ≥65 years, %	11.6	14.3	0.08	13.1	16.1	0.09
Men, %	73.3	73.3	-	69.8	69.8	-
Median annual income, 10^4 yen	336	336	0.007	336	336	0.007
BMI, kg/m^2	25.1 (4.2)	25.1 (4.2)	0.00	25.3 (4.3)	25.3 (4.4)	0.00
LDL-cholesterol, mg/dL	128.0 (34.2)	121.1 (31.1)	-0.21	129.8 (34.6)	121.2 (31.4)	-0.26
HDL-cholesterol, mg/dL	60.1 (17.0)	59.9 (17.0)	-0.01	61.5 (17.6)	61.4 (17.5)	-0.01
HbA1c, %	5.9 (1.1)	5.9 (0.8)	0.0	5.9 (1.0)	5.8 (0.8)	-0.11
eGFR, mL/min/1.73m^2	76.6 (15.7)	75.4 (15.9)	-0.08	75.2 (15.2)	74.2 (15.5)	-0.07
Current smoking, %	35.1	32.5	-0.05	34.1	31.2	-0.07
Current drinking, %	39.3	37.8	-0.03	37.3	35.8	-0.03
Receiving antihypertensive treatment, %	0.0	100.0	-	0.0	100.0	-
Diabetes, %	9.8	14.1	0.13	9.5	13.9	0.14
Receiving antidiabetic treatment, %	6.8	12.6	0.20	6.4	12.0	0.19
Dyslipidemia, %	59.2	60.8	0.03	60.5	61.5	0.02
Receiving lipid-lowering treatment, %	8.7	21.5	0.36	9.9	23.8	0.38
History of stroke, %	1.9	4.0	0.12	1.6	3.6	0.13
History of cardiovascular disease, %	4.8	8.4	0.15	4.1	7.4	0.14
History of kidney disease, %	0.7	1.3	0.06	0.7	1.2	0.05
Proteinuria, %	9.8	6.9	-0.10	7.7	5.6	-0.08
Interval between health check-ups, years	-	1.1 (0.3)	-	-	1.2 (0.6)	-
Median [interquartile range], years	-	1.0 (0.98–1.03)	-	-	1.0 (0.98–1.08)	-
5th–95th percentile, years	-	0.8–1.5	-	-	0.8–2.1	-
Corporate entity type: Corporation, %	98.9	98.9	-	98.9	98.9	-
Corporate entity type: Self-employed, %	1.1	1.1	-	1.1	1.1	-
Industry: Primary, %	0.9	0.9	-	1.0	1.0	-
Industry: Secondary, %	12.5	12.5	-	12.0	12.0	-
Industry: Tertiary, %	86.6	86.6	-	87.0	87.0	-
SBP, mm Hg	147.8 (21.0)	134.0 (16.8)	-0.73	148.6 (20.5)	134.1 (16.7)	-0.78
DBP, mm Hg	92.1 (14.2)	83.2 (11.4)	-0.69	92.5 (14.0)	83.1 (11.4)	-0.74
SBP/DBP <120/ <80 mmHg, %	6.2	14.9	0.29	5.4	14.7	0.31
SBP/DBP 120–129/ <80 mmHg, %	5.4	11.7	0.23	5.0	12.1	0.26
SBP/DBP 130–139/ 80–89 mmHg, %	17.8	32.5	0.34	17.0	32.7	0.37
SBP/DBP 140–159/ 90–99 mmHg, %	31.5	29.8	-0.04	31.9	29.6	-0.05
SBP/DBP 160–179/ 100–109 mmHg, %	25.8	9.0	-0.45	27.5	8.8	-0.50
SBP/DBP ≥180/ ≥110 mmHg, %	13.4	2.2	-0.43	13.3	2.1	-0.43

Abbreviations: B, unstandardized regression coefficient; SE, standard error; BMI, body mass index; JSH 2019, 2019 Japanese Society of Hypertension Guidelines; LDL, low-density lipoprotein; Partial R^2, partial coefficient of determination; SBP, systolic blood pressure; SE, standard error.

Supplementary Table 2. In the multiple linear regression model, systolic BP at the post-treatment health checkup was the dependent variable.

**Variable**	B	SE	t-value	Partial R^2, %	P-value
Male sex (1 = male, 0 = female)	1.57	0.03	48.30	0.18	<.0001
Age, per 1-year increase	0.13	0.00	81.35	0.50	<.0001
BMI, per 1 kg/m² increase	0.39	0.00	115.28	1.00	<.0001
LDL, per 1 mg/dL increase	0.02	0.00	49.17	0.18	<.0001
HDL, per 1 mg/dL increase	0.07	0.00	80.13	0.49	<.0001
Receiving antidiabetic treatment	1.13	0.04	25.96	0.05	<.0001
Receiving lipid-lowering treatment	-0.92	0.03	-29.06	0.06	<.0001
Current smoking	-0.23	0.03	-8.01	0.00	<.0001
Current alcohol drinking	0.90	0.03	29.48	0.07	<.0001
Interval between health check-ups, per 1-year increase	0.22	0.03	8.78	0.01	<.0001
Month of pre-treatment health check-up: February	-0.02	0.08	-0.26	0.00	0.80
Month of pre-treatment health check-up: March	0.29	0.09	3.31	0.00	0.00
Month of pre-treatment health check-up: April	0.32	0.10	3.27	0.00	0.00
Month of pre-treatment health check-up: May	0.58	0.09	6.45	0.00	<.0001
Month of pre-treatment health check-up: June	0.65	0.09	7.50	0.00	<.0001
Month of pre-treatment health check-up: July	0.79	0.09	9.19	0.01	<.0001
Month of pre-treatment health check-up: August	0.76	0.09	8.78	0.01	<.0001
Month of pre-treatment health check-up: September	0.50	0.08	6.02	0.00	<.0001
Month of pre-treatment health check-up: October	0.15	0.08	1.80	0.00	0.07
Month of pre-treatment health check-up: November	0.23	0.08	2.91	0.00	0.00
Month of pre-treatment health check-up: December	-0.11	0.08	-1.33	0.00	0.18
Month of post-treatment health check-up: February	-0.09	0.08	-1.15	0.00	0.25
Month of post-treatment health check-up: March	-0.79	0.09	-9.20	0.01	<.0001
Month of post-treatment health check-up: April	-0.36	0.10	-3.69	0.00	0.00
Month of post-treatment health check-up: May	-1.45	0.09	-16.16	0.02	<.0001
Month of post-treatment health check-up: June	-2.19	0.09	-25.68	0.05	<.0001
Month of post-treatment health check-up: July	-2.49	0.08	-29.43	0.07	<.0001
Month of post-treatment health check-up: August	-2.35	0.09	-27.54	0.06	<.0001
Month of post-treatment health check-up: September	-1.55	0.08	-18.78	0.03	<.0001
Month of post-treatment health check-up: October	-0.61	0.08	-7.73	0.00	<.0001
Month of post-treatment health check-up: November	-0.26	0.08	-3.27	0.00	0.00
Month of post-treatment health check-up: December	-0.22	0.08	-2.70	0.00	0.01
Pre-treatment SBP, per 1 mmHg increase	0.36	0.00	567.04	19.61	<.0001
Annual income, per 100,000 yen increase	-0.02	0.00	-50.42	0.19	<.0001
Post-JSH 2019 Guideline Publication Period	0.34	0.03	10.41	0.01	<.0001
Industry: Primary	0.31	0.13	2.37	0.00	0.02
Industry: Secondary	0.18	0.04	4.61	0.00	<.0001
Corporate Type: Self-employed	-0.10	0.12	-0.83	0.00	0.41
HbA1c ≥ 6.5%	1.54	0.06	23.78	0.04	<.0001
eGFR < 60 mL/min/1.73 m²	-2.16	0.04	-49.19	0.18	<.0001
No history of stroke	0.88	0.06	13.62	0.01	<.0001
No history of cardiovascular disease	1.25	0.05	25.06	0.05	<.0001
No history of kidney disease	-2.72	0.08	-34.81	0.09	<.0001
Negative proteinuria	-3.34	0.06	-51.66	0.20	<.0001

Missing values for covariates were treated as a separate category in the model.

Abbreviations: B, unstandardized regression coefficient; SE, standard error; BMI, body mass index; JSH 2019, 2019 Japanese Society of Hypertension Guidelines; LDL, low-density lipoprotein; Partial R^2, partial coefficient of determination; SBP, systolic blood pressure; SE, standard error.

Supplementary Table 3. Multiple linear regression analysis of the association among healthcare resource indicators, Hospital Pharmacist Maldistribution Index, and prefecture-level BP control rates

Prefecture-Level Indicator	B	SE	t-value	P-value
Hospital Pharmacist Maldistribution Index, per 1-point increase	7.535	2.313	3.26	0.0023
Outpatient ABPM Claims, per 1 claim per 100,000 population	0.012	0.014	0.89	0.38
Average Daily Number of Outpatients, per 1 person per 100,000 population	0.003	0.003	0.99	0.33
Health Insurance Premium Rate, per 1% increase	0.061	0.789	0.08	0.94
Participation Rate in Specific Health Check-ups, per 1% increase	-0.020	0.040	-0.50	0.62
Number of General Hospital Beds, per 1 bed per 100,000 population	-0.005	0.003	-1.34	0.19

This table shows the results of the weighted multiple linear regression analysis. The dependent variable was the prefecture-level-adjusted proportion of individuals achieving a systolic/diastolic blood pressure of <130/<80 mmHg by prefecture (Figure 2). All six indicators were simultaneously entered into the model. The analysis was weighted according to the number of participants in each prefecture.

Abbreviations: B, unstandardized regression coefficient; SE, standard error; ABPM, ambulatory blood pressure monitoring.

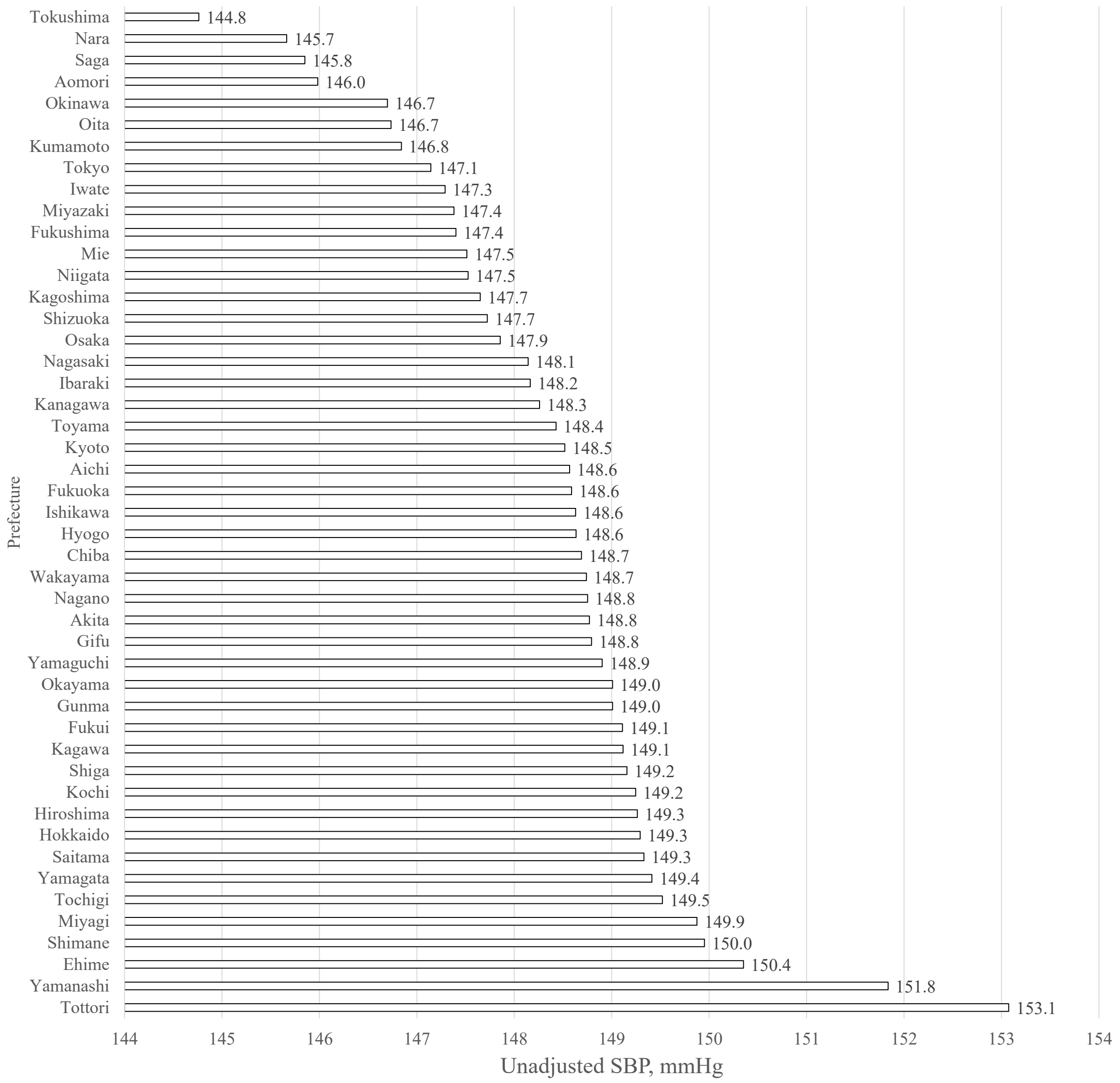

Supplementary Figure 1. Unadjusted SBP (mmHg) at the pre-treatment health check-up by prefecture

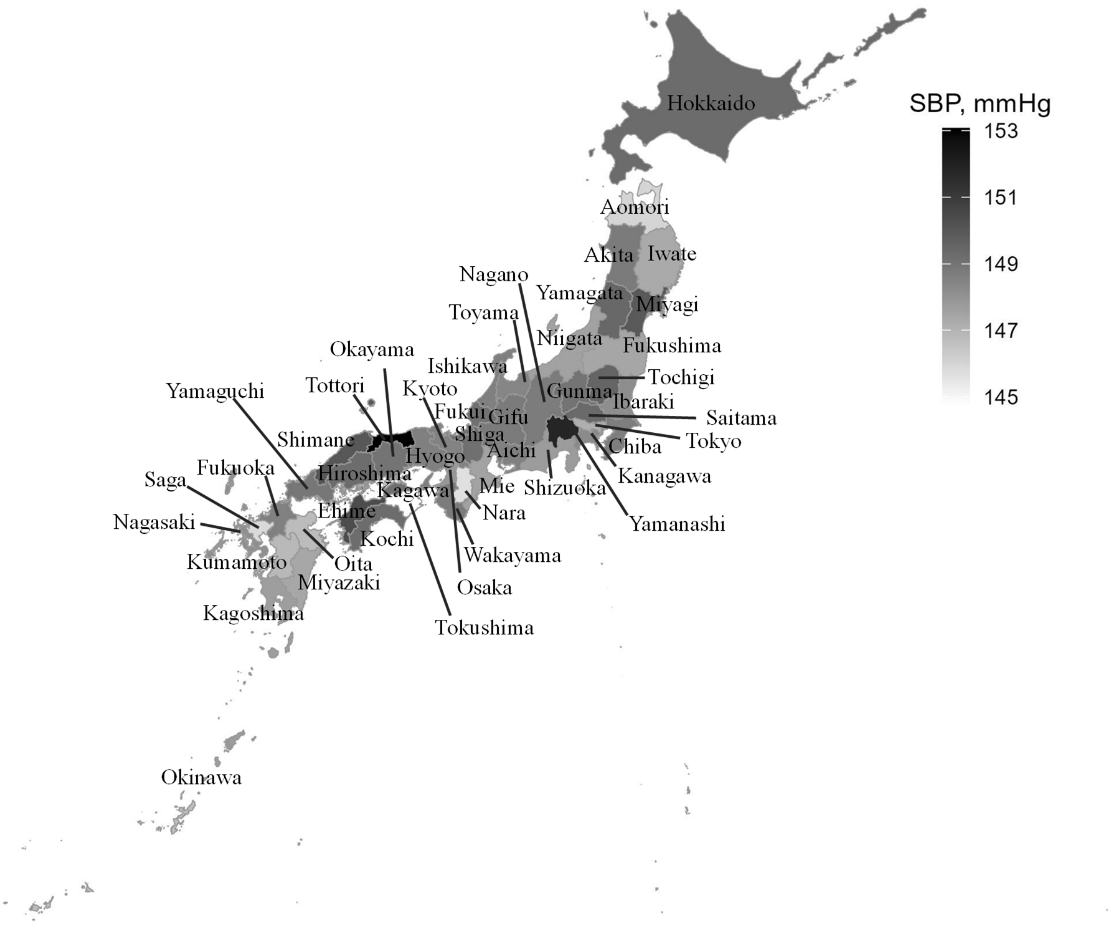

Supplementary Figure 2. Geographic distribution of unadjusted SBP (mmHg) at the pre-treatment health check-up by prefecture

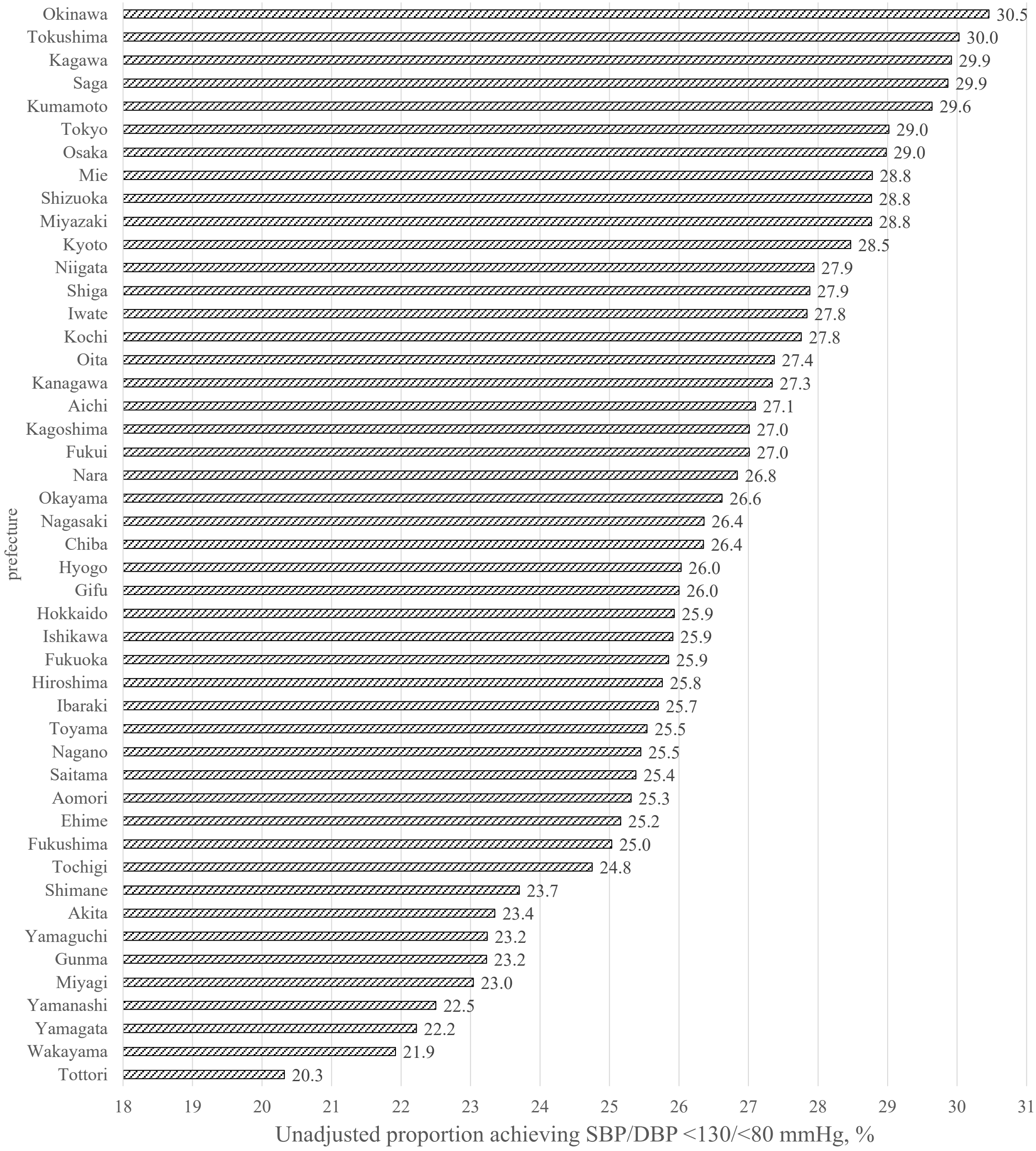

Supplementary Figure 3. Unadjusted proportion of individuals achieving SBP/DBP <130/<80 mmHg at the post-treatment health check-up (%) by prefecture

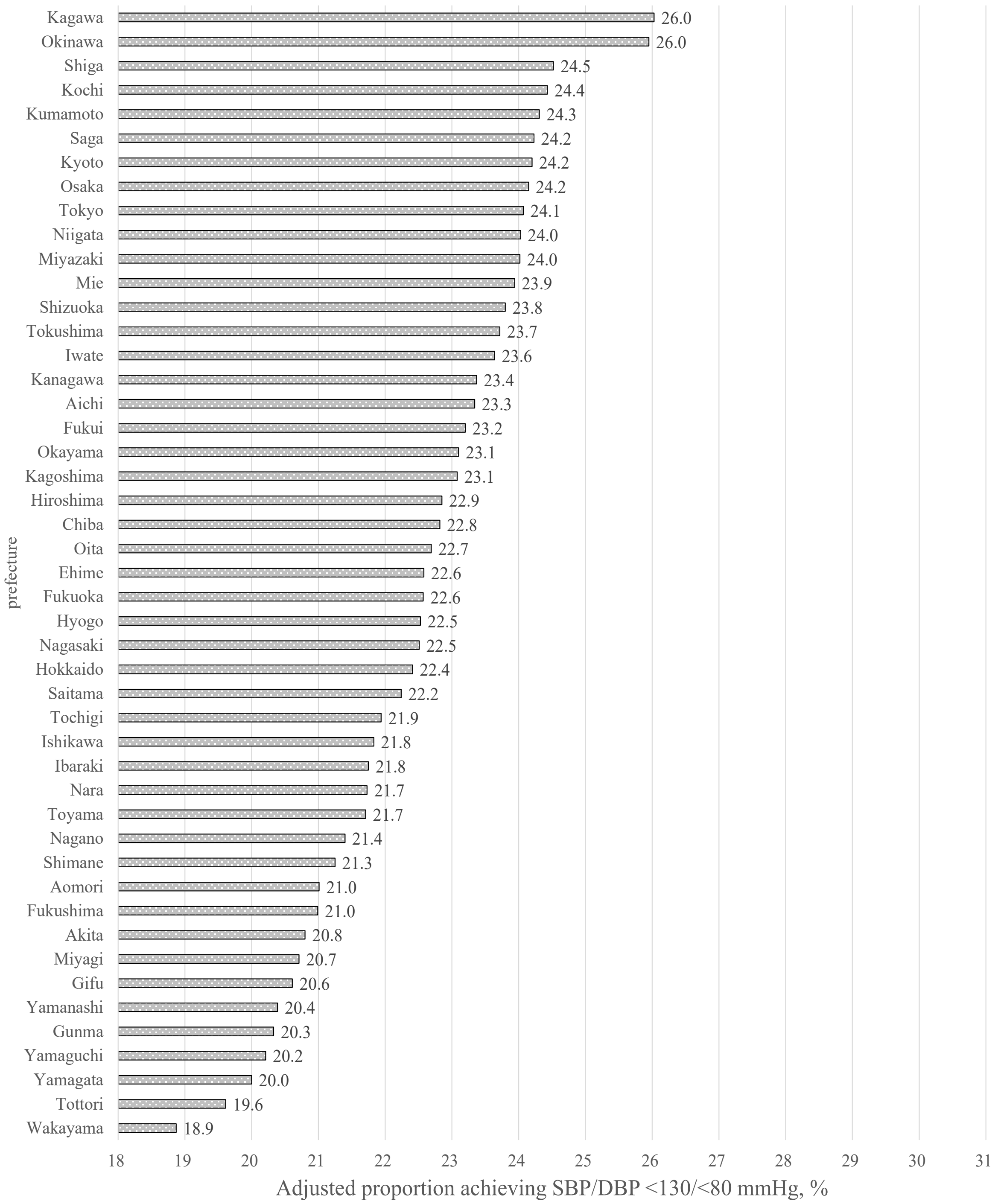

Supplementary Figure 4. Adjusted proportion of individuals achieving SBP/DBP <130/<80 mmHg at the post-treatment health check-up (%) by prefecture, estimated using multiple imputation.

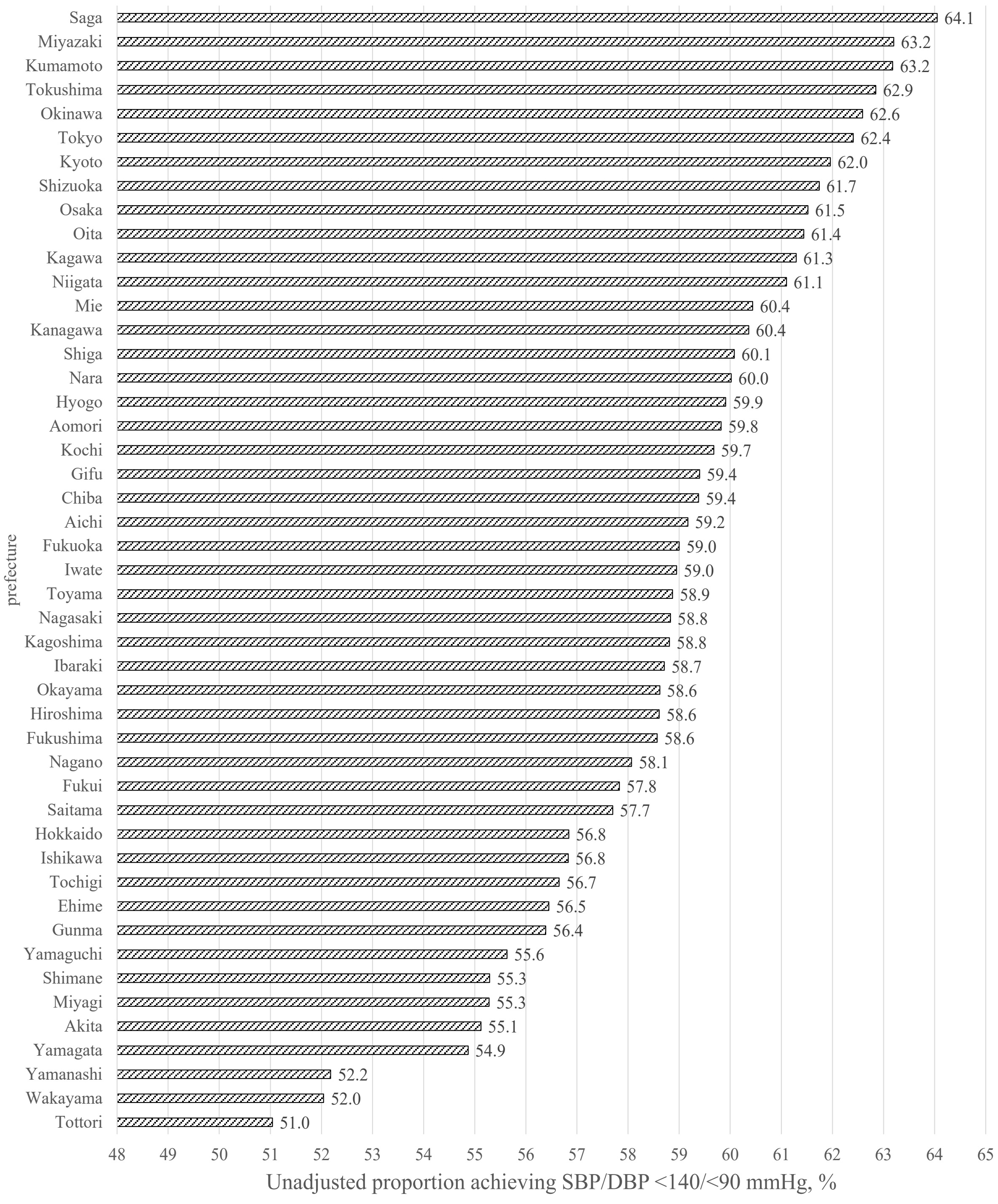

Supplementary Figure 5. Unadjusted proportion of individuals achieving SBP/DBP <140/<90 mmHg at the post-treatment health check-up (%) by prefecture

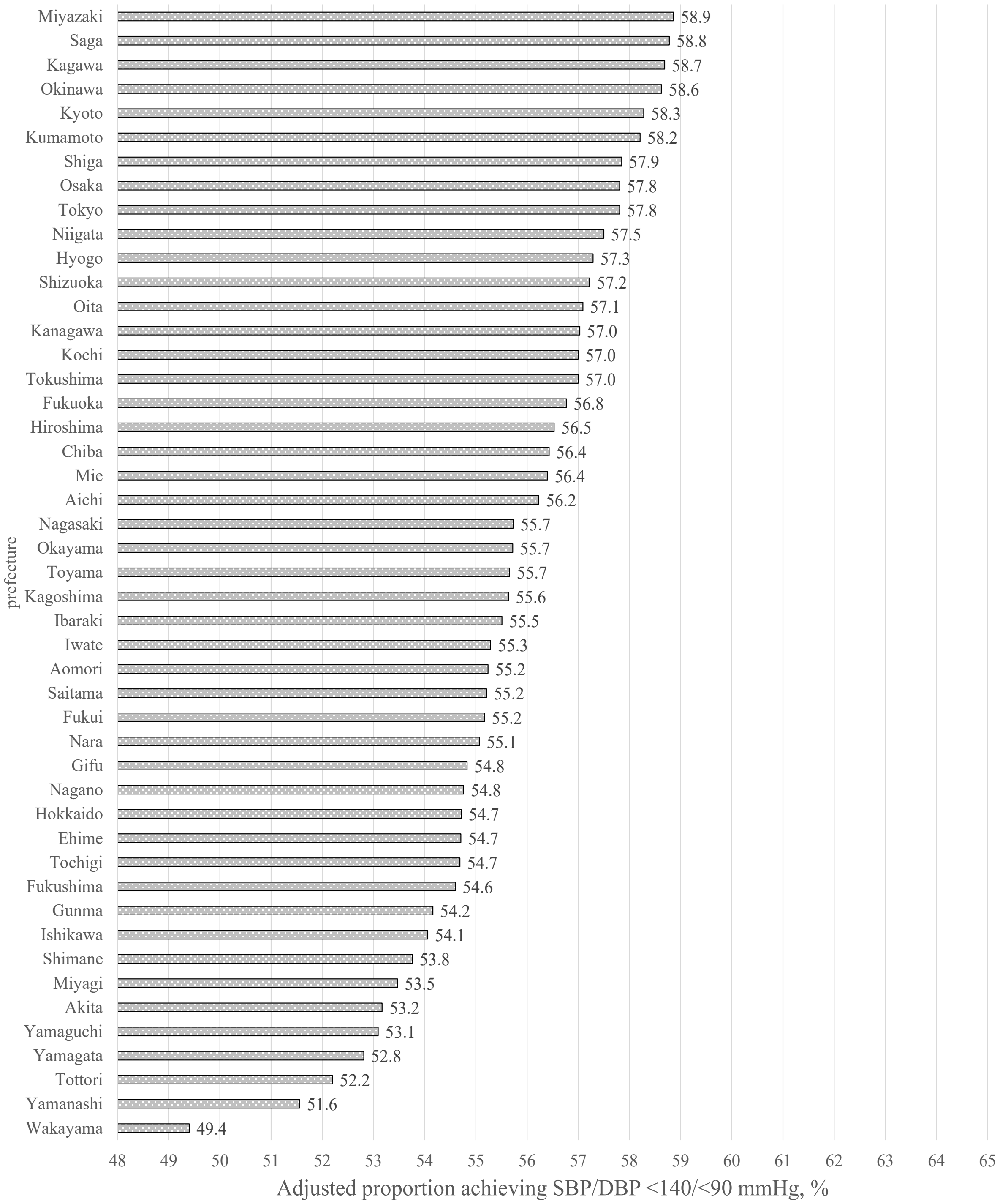

Supplementary Figure 6. Adjusted proportion of individuals achieving SBP/DBP <140/<90 mmHg at the post-treatment health check-up (%) by prefecture

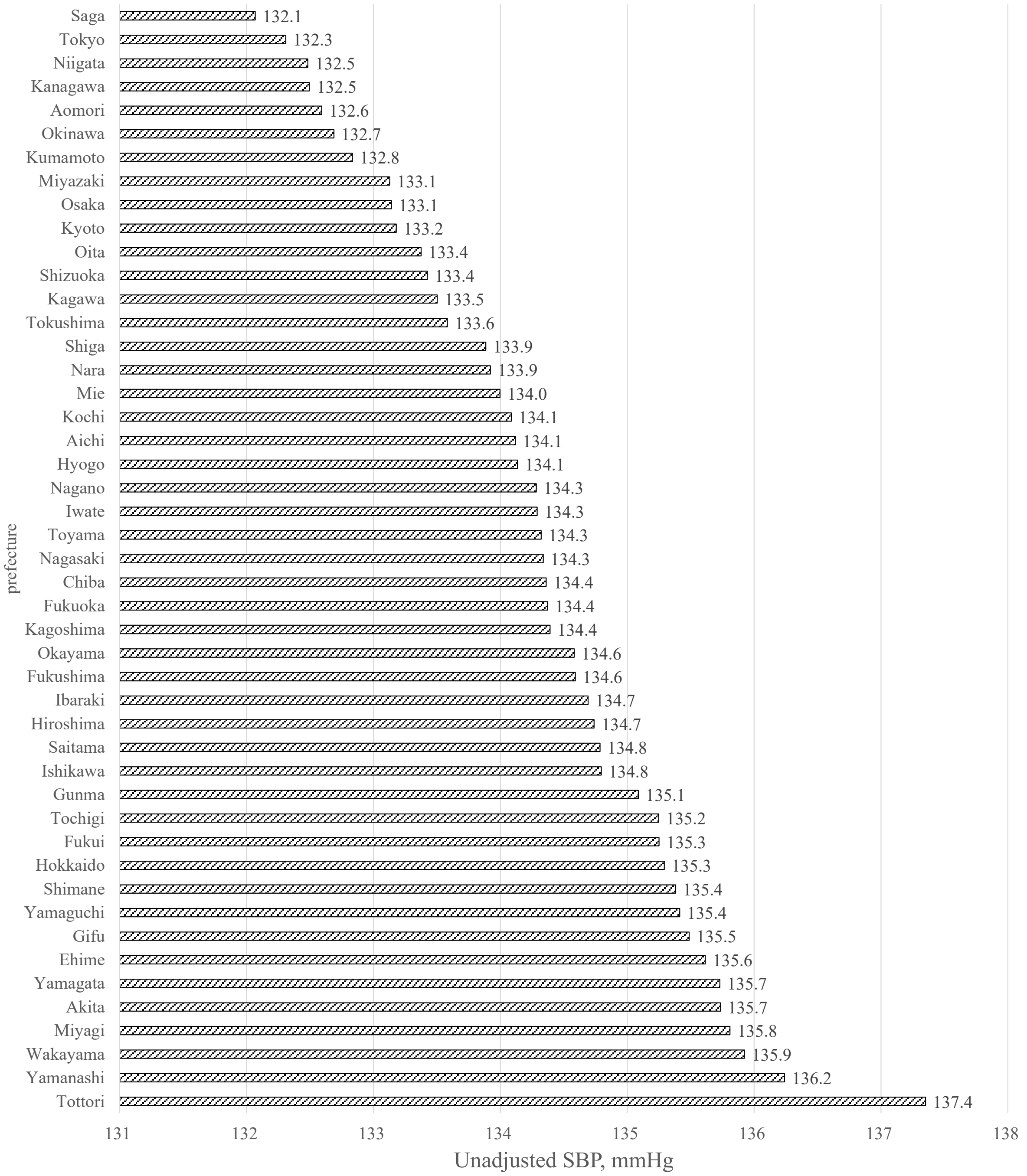

Supplementary Figure 7. Unadjusted SBP (mmHg) at the post-treatment health check-up by prefecture

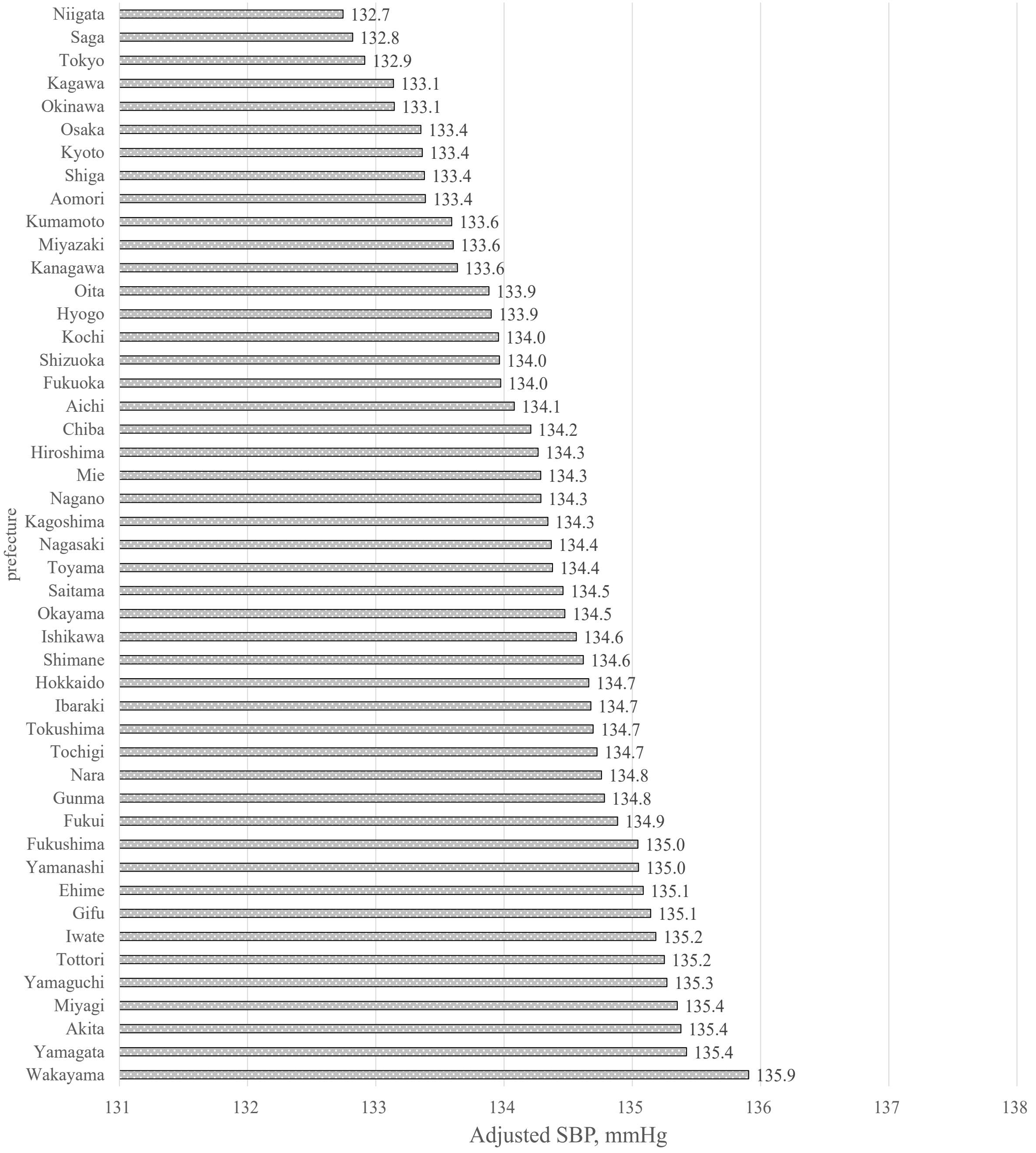

Supplementary Figure 8. Adjusted SBP (mmHg) at the post-treatment health check-up by prefecture

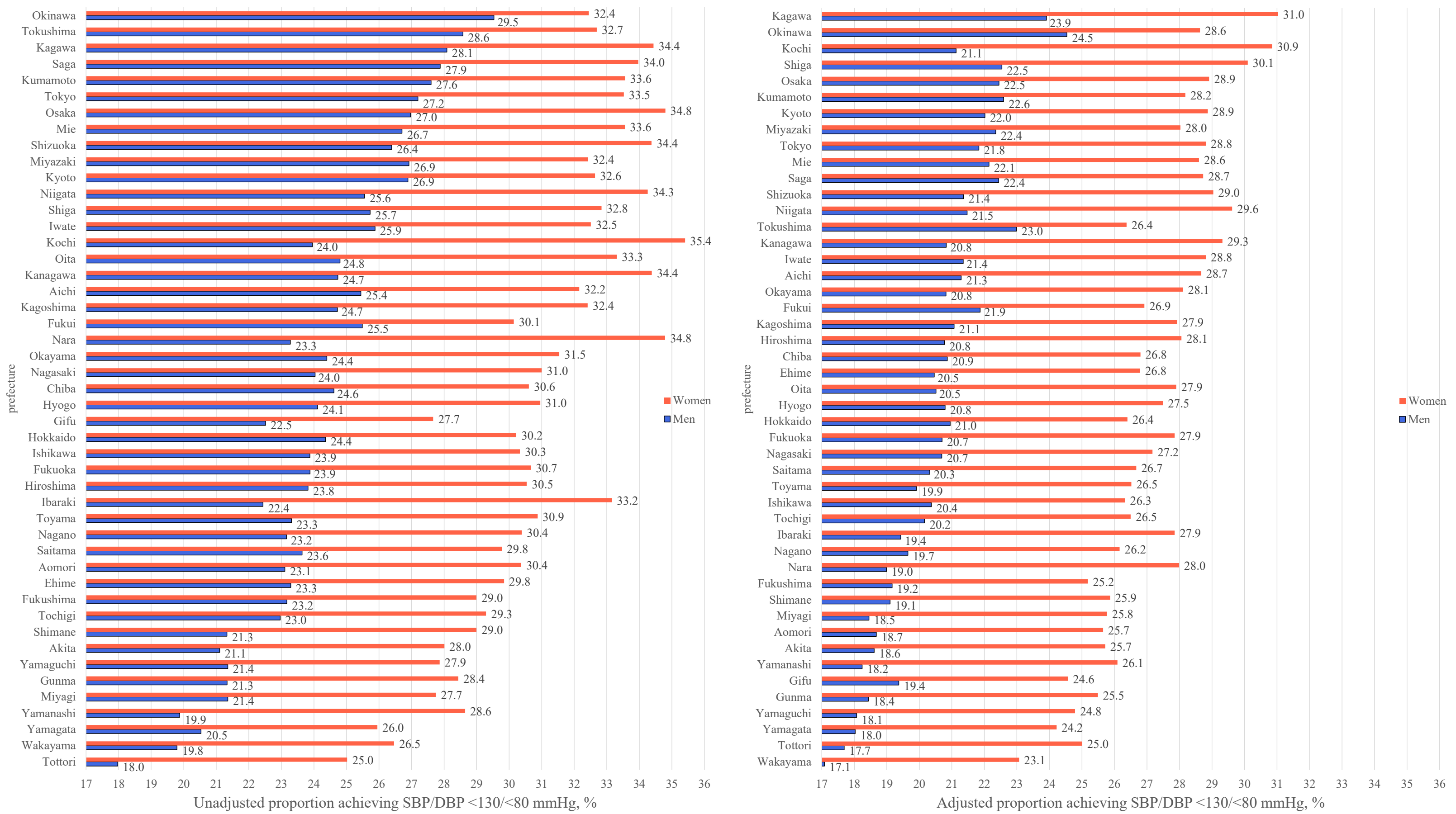

Supplementary Figure 9. Unadjusted (left) and adjusted (right) proportion of men and women achieving SBP/DBP <130/<80 mmHg at the post-treatment health check-up (%) by prefecture

Prefectures are arranged in descending order of the BP control proportion for the total population (men and women combined).

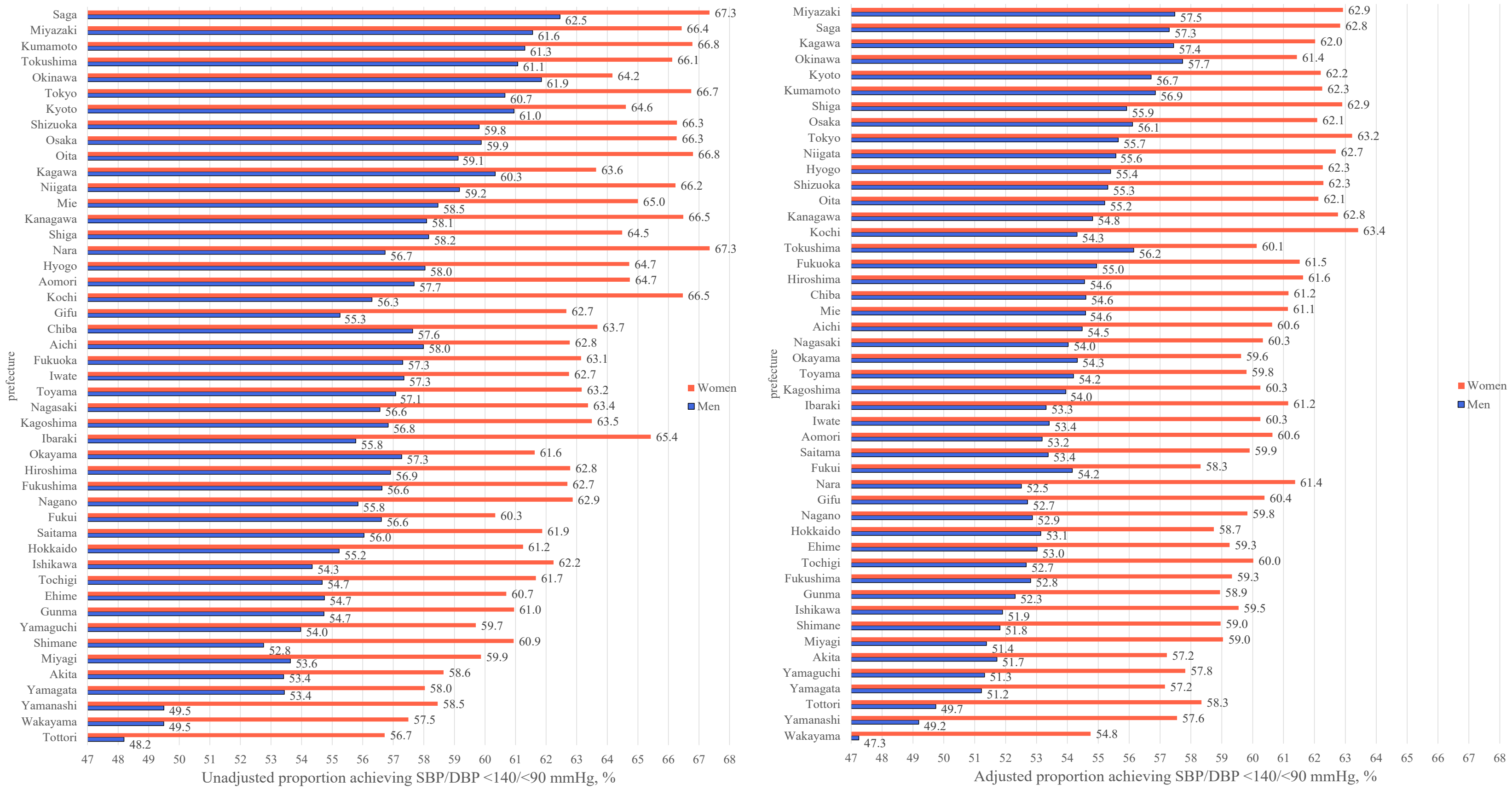

Supplementary Figure 10. Unadjusted (left) and adjusted (right) proportion of men and women achieving SBP/DBP <140/<90 mmHg at the post-treatment health check-up (%) by prefecture

Prefectures are arranged in descending order of the BP control proportion for the total population (men and women combined).

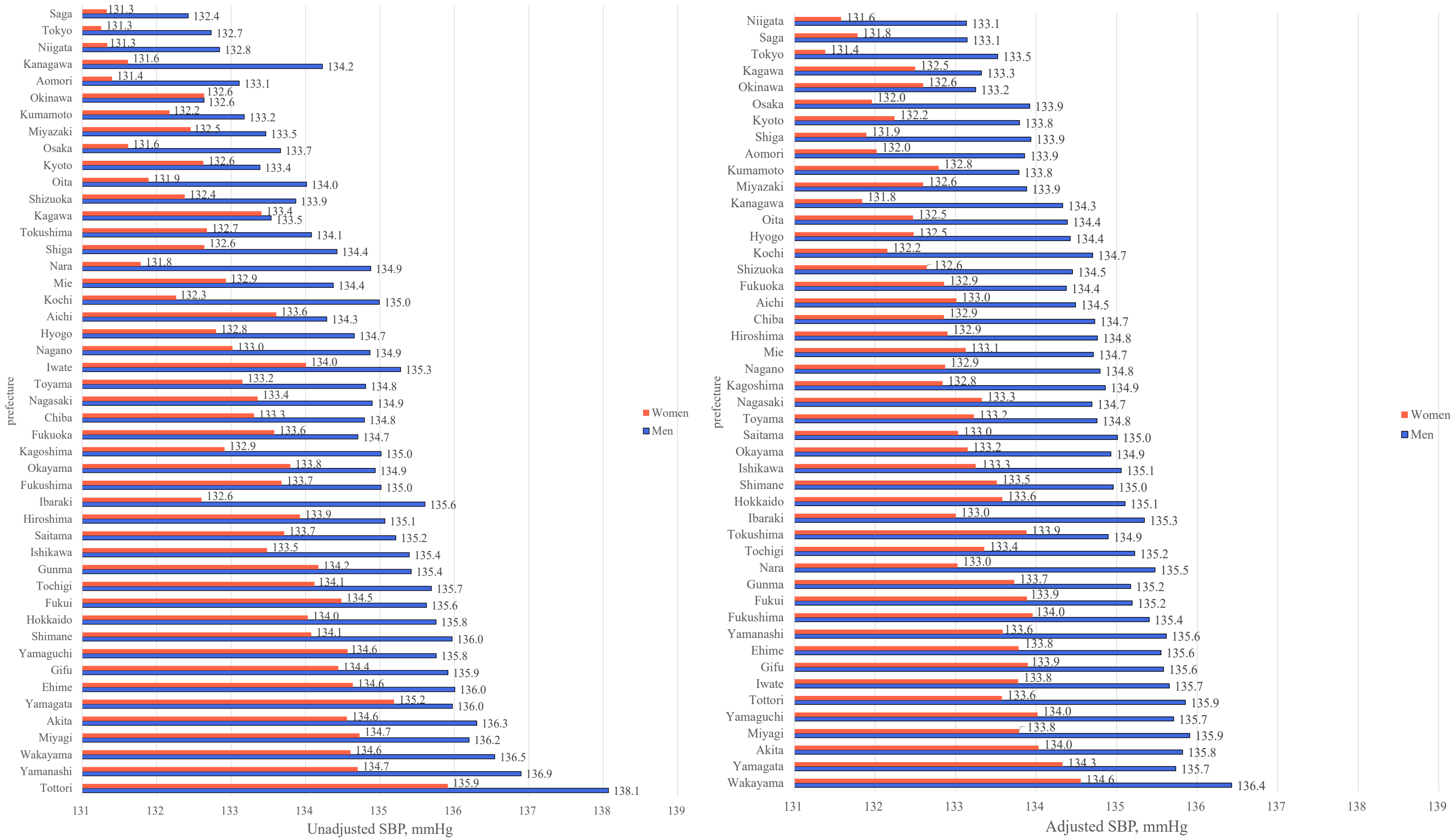

Supplementary Figure 11. Unadjusted (left) and adjusted (right) SBP (mmHg) values for men and women at the post-treatment health check-up by prefecture

Prefectures are arranged in ascending order of the SBP for the total population (men and women combined).

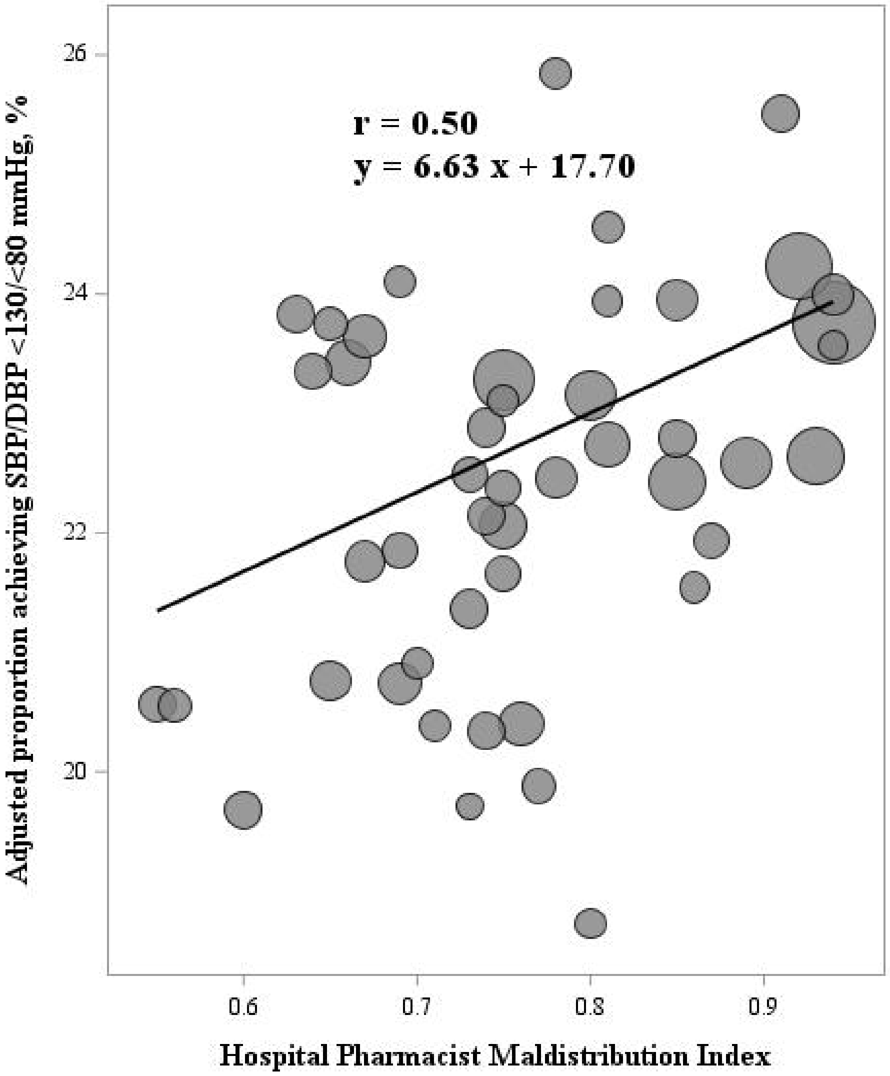

Supplementary Figure 12. Scatter plot between the adjusted proportion of individuals with systolic/diastolic BP <130/<80 mmHg and the Hospital Pharmacist Maldistribution Index across prefectures

This scatter plot shows the ecological association between the Hospital Pharmacist Maldistribution Index and the prefecture-level BP control rate. Each circle represents one of Japan's 47 prefectures, with its size proportional to the number of study participants from that prefecture. The y-axis shows the adjusted proportion of individuals BP control to <130/<80 mmHg, which is the same data presented in Figure 2. The x-axis shows the Hospital Pharmacist Maldistribution Index. The regression line and Pearson's correlation coefficient (r) are derived from a simple linear regression analysis weighted by the number of participants.
